# Sequence Variants Linked to Key Traits in Interspecific Crosses between African and Asian Rice

**DOI:** 10.3390/plants9121653

**Published:** 2020-11-26

**Authors:** Hayba Badro, Marie-Noelle Ndjiondjop, Agnelo Furtado, Robert Henry

**Affiliations:** 1 Queensland Alliance for Agriculture and Food Innovation, University of Queensland, Brisbane, QLD 4072, Australia; haybaq@yahoo.com (H.B.); a.furtado@uq.edu.au (A.F.); 2 Department of Biotechnology, College of Science, University of Baghdad, Baghdad, Iraq; 3 Africa Rice Center (AfricaRice), M’bé Research Station, Bouake 01 BP 2551, Cote d’Ivoire; m.ndjiondjop@gmail.com

**Keywords:** *Oryza sativa*, *Oryza glaberrima*, heading date, tiller number, 1000 grain weight, trait-associated variants (TAVs)

## Abstract

Asian and African rice gene pools vary in many traits that are important in rice breeding. The genetic basis of these differences was evaluated by analysis of important agronomic traits in crosses between African and Asian rice. Trait-associated variants (TAVs) influencing three quantitative agronomic traits, heading date (Hd), tiller number at maturity (T), and 1000 grain weight (TGW), were identified by association analysis of crosses between Asian and African rice. Populations were developed by crossing WAB56-104 (*Oryza sativa*) and CG14 (*Oryza glaberrima*). DNA from plants with extremely high or low values for these phenotypes was bulked and sequenced. The reference genome of *O. sativa* cv *Nipponbare* was used in general association analysis and candidate gene analysis. A total of 5152 non-synonymous single nucleotide polymorphisms (SNPs) across 3564 genes distinguished the low and the high bulks for Hd, T, and TGW traits; 611 non-synonymous SNPs across 447 genes were found in KEGG pathways. Six non-synonymous SNPs were found in the sequences of LOC107275952, LOC4334529, LOC4326177, LOC107275432, LOC4335790, and LOC107275425 genes associated with Hd, T, and TGW traits. These genes were involved in: abscisic-acid biosynthesis, carotenoid biosynthesis, starch and sucrose metabolism, and cytokinin biosynthesis. Analysis of 24 candidate genes associated with Hd, T, and TGW traits showed seven non-synonymous variations in the sequence of *Hd3a* and *Ehd2* from the Hd genes (not in a KEGG pathway), *D10* and *D53* from the T genes (strigolactones biosynthetic pathway), and *Gn1a* and *GIF1* from the TGW genes (cytokinin biosynthetic and starch and sucrose metabolism pathways). This study identified significant differences in allele frequencies supported by high sequence depth in analysis of bulks displaying high and low values for these key traits. These trait-associated variants are likely to be useful in rice improvement.

## 1. Introduction

Rice is one of the world’s most important cereal crops and the staple food for more than half of the world’s population [1]. The rapid escalation of the world population over the next thirty years requires an increase in the production of cereal grains, including rice, to meet food demand. Increased production may be achieved by improvements in agronomically important traits through introgression or hybridisation. Key traits include heading date (Hd) (flowering time) and grain yield, which is determined by tiller number (T), 1000 grain weight (TGW), and grain number (Gn) [2,3,4,5]. Numerous recent studies have reviewed in detail the available information on these traits for use in plant breeding [6,7,8,9,10].

One of the early crop domestication traits studied was flowering time or heading date for rice. Extensive studies have established that the heading date is affected by temperature and photoperiod (day length) [11] and controlled by multiple genes, including heading date (*Hd*), early heading date (*Ehd*), and days to heading (*DTH*) genes, within two photoperiod pathways, which are named on the basis of the key genes, heading date 1 (*Hd1*) [12] and early heading date 1 (*Ehd1*) [13,14]. The key genes of flowering time involved in the Hd1 photoperiod pathway are conserved between *Arabidopsis thaliana* and rice, including heading date 1 (*Hd1*) and heading date 3a (*Hd3a*) [14,15]. In addition to the Hd1 photoperiod pathway, there are other genes uniquely regulating flowering time in cultivated rice through the Ehd1 pathway, including the main gene (*Ehd1*), down-regulator genes (*Ghd7*, *Hd5*, *Hd2* and *Hd16*) [2,16,17,18], and up-regulator genes (*Ehd2* and *Ehd3*) [19,20], that have no homologs in the genome of *Arabidopsis* [13,14,15]. Although the genetic and molecular flowering pathway is relatively well understood, the mechanisms controlling flowering time diversity have been unclear. Many previous studies have analysed the sequences of the candidate genes and their expressions to evaluate their contributions to the heading date diversity in cultivated rice [4,21], leading to the suggestion that variations in *Hd1*, *Ehd1*, and *Hd3a* determine this diversity.

Tiller number is one of the major agronomic traits that can determine plant architecture and grain yield. This trait is regulated by a number of hormones, including auxin, cytokinin (CK), and strigolactones (SLs), where auxin and SLs act as a repressor and can inhibit shoot branching (tillering), unlike cytokinin, which stimulates tillering [22,23,24,25]. A set of *Dwarf* genes are involved in the strigolactones biosynthesis pathway, including *D27*, *D17* (*HTD1*), and *D10* [25,26]. Moreover, the SLs-signalling pathway also affects the regulation of axillary bud outgrowth. This pathway includes more *Dwarf* genes, named *D3* and *D14* [27]. Besides the *Dwarf* genes, additional rice tiller-regulating genes have been identified and characterised, such as the reduced culm number (*RCN*), MONOCULM (*MOC*), and TEOSINTE BRANCHED (*OsTB*) genes [6]. Among a serial of *RCN* genes, there is the *RCN1* gene, which positively regulates shoot branching [28]. The *MOC1* gene is one of the three identified *MOC* genes required for the formation of tiller buds and promotion of their outgrowth [25,29]. An increase in the expression of the *MOC1* gene promotes excessive shoot branching. In contrast, overexpression of the *OsTB1* gene suppress the outgrowth of axillary buds [24,30].

Grain weight, measured as 1000 grain weight (TGW), and grain number (Gn) along with tiller number are the key components that contribute greatly to the grain yield, and they are regulated through some plant hormones such as cytokinin and abscisic-acid, and several genes, including grain productivity (*Gn1a*), grain width (*GW2* and *GW5*), grain size/shape (*GS3*), and grain incomplete filling (*GIF1*) genes [2,3,10,31]. The *Gn1a* plays a pivotal role in cytokinin metabolism pathways. Cytokinin is one of the major plant growth phytohormones that controls most aspects of plant development [31,32]. The *GIF1* gene also acts as one of the important domestication genes that has been shown to be significantly related to the grain weight trait due to regulation of the levels of sugar for starch synthesis [10]. In addition, another plant hormone influences grain filling, Abscisic-Acid (ABA), which is a carotenoid-derivative [33]. Moreover, grain width and grain size, which largely define grain weight trait, were suggested to be negatively regulated by *GW2* and *GS3* genes [3,34,35].

There are a number of approaches for identification of trait-associated variants, one of which is genome-wide association (GWA). GWA is a tool that inspects the entire genome to investigate the contribution of genetic variations to specific phenotypic traits [36,37]. To detect the genes that control certain complex traits, even small-effect genes, and provide a higher-resolution mapping of trait-associated variants (TAVs) through GWAS, genotyping is required for every member of the population [38,39]. Therefore, GWAS may be laborious and costly, especially for large populations of species with large genomes, even when using the most cost-effective high-throughput sequencing techniques such as genotyping-by-sequencing [40]. An alternative approach for rapid identification of genotype-phenotype correlations is bulk segregant analysis (BSA), which identifies any type of molecular marker genetically linked to the causal gene [41]. BSA was developed by Michelmore et al. [42] to reduce the cost and the workload of genotyping every individual of the population by several fold; this approach allows genotyping pools of individuals sorted by phenotype into two extreme groups from a large population. In combination with next-generation sequencing (NGS) techniques, BSA has become a powerful tool for genetic analysis, termed NGS-based BSA, particularly with the ongoing improvement of NGS procedures and the rapid reduction in costs [39,43,44].

The African rice varieties have adaptive or protective mechanisms of resistance to major biotic and abiotic stresses, such as drought, iron toxicity, weed, nematodes, African rice gall midge [45,46], and bacterial blight [47]. However, African rice is generally characterized by undesirable agronomic traits, including lodging, limited number of spikelets per panicle due to few secondary branches, grain shattering, and long seed dormancy [48]. To combine traits of economic importance from both the Asian and the African rice, interspecific breeding programs were initiated by the Africa Rice Center (www.africarice.org) in the early 1990s, which resulted in the development and the release of New Rice for Africa (NERICA) varieties. Most NERICA varieties showed a combination of the high productivity of *O. sativa* with the hardiness of *O. glaberrima*. First sets of NERICAs were developed from a cross between an upland *O. sativa* tropical japonica variety (such as WAB56-104, WAB56-50, and WAB181-18) as the recipient parent, and an *O. glaberrima* variety, CG14, as the donor parent [49].

There is a large pool of genetic variation available in landraces of the two cultivated rice species *O. sativa* and *O. glaberrima*. These resources are known to contain many interesting traits for breeding, including good to strong tolerance to abiotic and biotic stresses as well as various nutritional and agronomical traits of interest. However, it is often difficult to utilize these natural sources of genetic diversity because of fertility barriers, linkage drag, and time and resources required to recover useful recombinants. The interspecific backcross lines are sister lines of NERICAs. They were developed using the same parents as NERICAs (e.g., CG14 and WAB56-104) to study this unexploited reservoir of useful genes with a focus on genomic regions related to heading date, tiller number, and 1000 grain weight.

The current study aimed to perform association analysis for three important agronomic traits in the African-Asian crossbreeding population in order to detect desirable alleles for rice breeding. For this purpose, we conducted association analysis by NGS-based BSA for three quantitative agronomic traits, heading date (Hd), tiller number at maturity (T), and 1000 grain weight (TGW), in a population of 285 individuals developed by crossbreeding between WAB56-104 (*O. sativa*) and CG14 (*O. glaberrima*), which had been analysed for several important agronomic traits, including these three traits. A pair of bulks was selected for each trait based on the phenotypic data, and the association analysis was then applied to 1) entire genome and 2) 24 candidate genes, separately, to determine the effective TAVs for Hd, T, and TGW traits.

## 2. Results

### 2.1. Phenotype Variation

**Figure 1:**
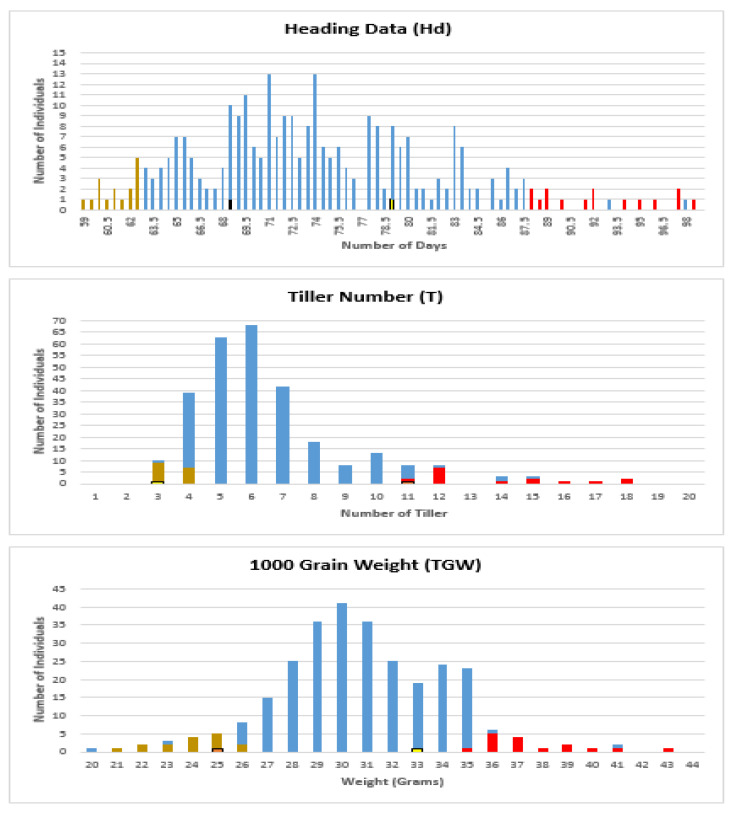
Frequency distribution of heading date (Hd), tiller number at maturity (T), and 1000 grain weight (TGW) traits within a population of 285 individuals. “X” represents the values of traits (days, tiller count, and gram, respectively) while “Y” represents individuals’ count. The selected individuals of two extreme phenotypic bulks, low and high, are marked in brown and red, respectively.

The distribution of each trait was examined (Figure 1) and used to define bulks for sequencing.

### 2.2. DNA Sequencing and Genotype Calling

Sequencing of the six bulks generated about 166 Gb of data containing 981 million of 151 bp paired-end reads, with sequence depth ranging between 68× and 89×. When raw data were trimmed at the quality limit of 0.01, an average of 14% of the read length and 10% of the number of reads was removed, thus reducing the data coverage to between 55× and 67×. Thereafter, about 81% of the trimmed reads, a total of 798 million reads, were mapped against the *O. sativa* cv Nipponbare (IRGSP-1.0, NCBI) genome; the coverage ranged between 41× and 54× at this stage (Table S1). When variants were called, the lowest and the highest number of variants, 8,365,429 and 20,332,280, were found in the low-bulk for the Hd trait (HdL) and the high-bulk for the tillering trait (TH), respectively. However, only 1–2% of the total number of variants represented the non-synonymous variants that have undergone further statistical analysis (Table S2).

### 2.3. Statistical Analysis of Marker-Trait Association

The number of non-synonymous variants was reduced further according to a zygosity (homozygous (hom) or heterozygous (het)) and frequency filter, which divided the non-synonymous SNPs into four groups, including hom-hom, hom-het, het-hom, and het-het, for all traits separately. In addition, the Chi-square test and manual checking contributed to this reduction significantly. For all traits, the hom-hom category included the lowest number of variants; in contrast, most SNPs were included in the het-het category. Tiller number trait resulted in the greatest number of non-synonymous SNPs (3375 SNPs in 2277 genes), followed by the TGW trait that generated 1479 non-synonymous SNPs in 1043 genes, whereas the Hd trait resulted in the least number of non-synonymous SNPs, 298 SNPs in 244 genes (Table S3).

### 2.4. Functional Annotation Analysis

**Figure 2:**
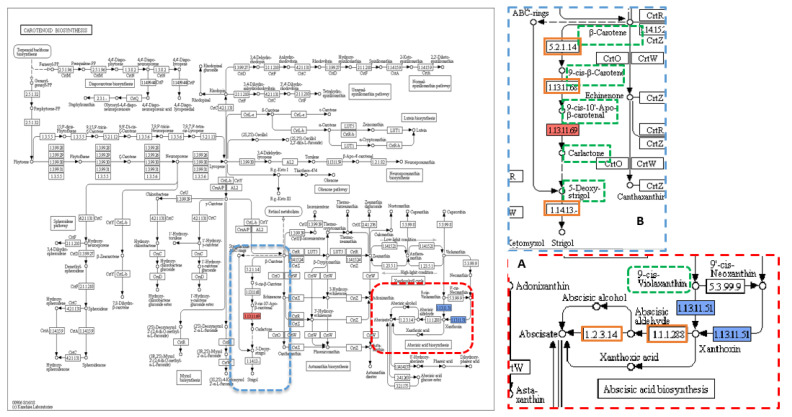
KEGG pathway of carotenoid biosynthesis containing (**A**) abscisic-acid (ABA), and (**B**) strigolactones (SLs) biosynthetic pathways. KEGG pathway of carotenoid biosynthesis containing (**A**) abscisic-acid (ABA) biosynthetic pathway (dotted red box) and (**B**) strigolactones (SLs) biosynthetic pathway (dotted blue box), dotted green squares: precursors and substrates included in ABA and SLs biosynthesis pathway, solid blue square: OsNCED5 (1.13.11.51-dioxygenase) enzyme encoded by LOC107275952 and/or LOC107275432 genes carrying the trait-associated variants (TAVs) identified in this study, solid red square: CDD8 (1.13.11.69-synthase) enzyme encoded by *D10* gene carrying the TAVs identified in this study, and orange squares: other enzymes involved in the pathway. Reprinted with permission from Kyoto Encyclopedia of Genes and Genomes (KEGG), http://www.kegg.jp/kegg/kegg1.html.

The annotations of 244, 2277, and 1043 genes that had variants significantly associated with the three traits Hd, T, and TGW, respectively, were then imported to Blast2GO. Of these genes, only 16, 311, and 120 genes were involved in KEGG pathways. KEGG pathways associated with the Hd trait involved 25 enzymes, and 16 enzymes were unique; biosynthesis pathway of antibiotics was recorded as the most common pathway (Table S4). Among the 16 genes, there were 18 SNPs distributed as follows: 1, 3, and 14 into hom-het, het-hom, and het-het categories, respectively. One non-synonymous SNP from the het-het category was found in the annotated sequence of the “LOC107275952” gene on chromosome 6, which was also known as “*NCED5*” and was present within the HdH-bulk at a frequency of 82% and coverage of 51× (Table 1). The *NCED5* gene encodes OsNCED5 (1.13.11.51-dioxygenase), which catalyses the key step in abscisic-acid (ABA) biosynthesis, where ABA is synthesised via the carotenoid pathway, which is one of 21 KEGG pathways found in this analysis (Figure 2A).

**Figure 3:**
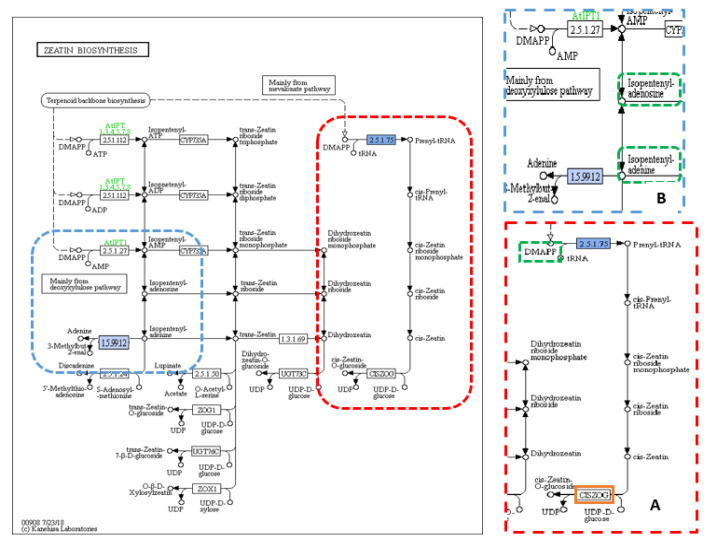
KEGG pathway of zeatin biosynthesis showing (**A**) cis-type cytokinin (CK) biosynthesis and (**B**) cytokinin (CK) signalling pathway. KEGG pathway of zeatin biosynthesis showing (**A**) cis-type cytokinin biosynthesis (dotted red box) and (**B**) cytokinin signalling pathway (dotted blue box), dotted green squares: the precursors and/or substrates included in CK biosynthesis pathway, solid blue square: (**A**) **2.5.1.75**–dimethylallyl transferase enzyme encoded by *IPT6* and/or *OsIPT4* (isopentenyl pyrophosphate transferases) (LOC107275425 and/or LOC4334529) genes carrying the TAVs identified in this study, and (**B**) cytokinin dehydrogenase 2 (CKX2) (1.5.99.12-dehydrogenase) enzyme encoded by *Gn1a* gene carrying the TAVs identified in this study, and orange squares: other enzymes involved in the pathway. Reprinted with permission from Kyoto Encyclopedia of Genes and Genomes (KEGG), http://www.kegg.jp/kegg/kegg1.html.

When the annotated sequences for the tillering trait were imported into Blast2GO, the results showed 97 KEGG pathways included only 311 sequences with 170 unique enzymes. Purine metabolisms was the most common pathway (Table S5). A total of 425 SNPs was found in 311 genes. The largest number of SNPs, 270, existed in the het-het category, followed by 88 SNPs in the hom-het group, and then 38 and 29 SNPs in het-hom and hom-hom categories, respectively. Among 97 KEGG pathways, there were two pathways, carotenoid biosynthesis and zeatin biosynthesis, with two enzymes, “1.13.11.69-synthase” and “2.5.1.75–dimethylallyl transferase”, related to the tillering trait. The carotenoid biosynthesis pathway included one annotated sequence of the “LOC4326177” gene (also known as *Dwarf 10* (*D10*)) (Figure 2B) that had one non-synonymous SNPs from the het-het category with a frequency of 11% in the TL-bulk and 86% in the TH-bulk at a coverage of 35× and 49×, respectively (Table 1). The second KEGG pathway was zeatin biosynthesis, which included one annotated sequence of the “LOC4334529” gene (also known as “Isopentenyl Pyrophosphate Transferases 4” (*OsIPT4*)) (Figure 3A) that had one non-synonymous SNPs from the het-hom category with a frequency of 14% in the TL-bulk and 96% in the TH-bulk at a coverage of 36× and 50×, respectively (Table 1).

**Figure 4:**
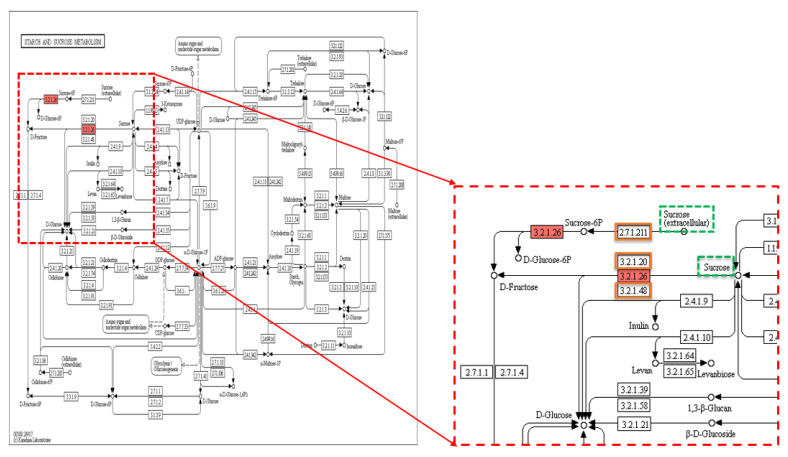
KEGG pathway of starch and sucrose metabolism showing sucrose metabolism. KEGG pathway of starch and sucrose metabolism showing sucrose metabolism (invertase catalyses sucrose into fructose and glucose) (dotted red box), dotted green squares: substrates including sucrose, solid red square: **3.2.1.26**-invertase enzyme encoded by *GIF1* (grain incomplete filling) (LOC4335790) gene carrying the TAVs identified in this study, and orange squares: other enzymes involved in the pathway. Reprinted with permission from Kyoto Encyclopedia of Genes and Genomes (KEGG), http://www.kegg.jp/kegg/kegg1.html.

Sixty-two KEGG pathways associated with the TGW trait involved 143 enzymes, with 82 enzymes being unique. Thiamine metabolism was recorded as the most common pathway (Table S6). There were 168 non-synonymous SNPs in 120 genes related to TGW, of which 12 SNPs were in the hom-het group, 16 SNPs in the het-hom group, and 140 SNPs in the het-het group. Out of 120 genes, there were three genes, LOC107275425 (*IPT6*), LOC107275432, and LOC4335790, present in three pathways, zeatin biosynthesis, carotenoid biosynthesis, and starch and sucrose metabolism (Figure 2A, Figure 3A and Figure 4), which were associated with grain filling and weight traits directly and indirectly [7,31,32,50]. Within the sequence of these three genes, LOC107275425 (*IPT6*), LOC107275432, and LOC4335790 that encode three enzymes, 2.5.1.75–dimethylallyl transferase, 1.13.11.51-dioxygenase, and 3.2.1.26-invertase, there were three non-synonymous SNPs found in the het-het group at a frequency of 80% in the 1000 grain weight low (TGWL)-bulk and 18% in the 1000 grain weight high (TGWH)-bulk, the het-hom category at a frequency of 29% in the TGWL-bulk and 97% in the TGWH-bulk, and the het-het group at a frequency of 84% in the TGWL-bulk and 18% in the TGWH-bulk, respectively. The coverage at the positions of these SNPs was 28× for the LOC107275432 gene in both bulks, 51× and 38× for the LOC107275425 (*IPT6*) gene, and 32× and 38× for the LOC4335790 gene in low and high bulks, respectively (Table 1). The first enzyme (2.5.1.75–dimethylallyl transferase) was involved in the biosynthesis of cis-type cytokinin, one of four types of cytokinin (Figure 3A), whereas the second enzyme (1.13.11.51-dioxygenase), which is also known as OsNCED5, acts as a key enzyme in the pathway of abscisic-acid biosynthesis via the carotenoid pathway (Figure 2A). Finally, the third enzyme (3.2.1.26-invertase), also known as cell-wall invertase, regulates the levels of sugar for starch metabolism during early grain-filling.

### 2.5. Analysis of Candidate Genes

A total of 72 annotated sequences of 24 genes associated with the three agronomic traits (Table S7) were successfully extracted, three annotated sequences for each gene, two from the genomes of the opposite bulks and one from the reference genome. SnapGene 4.0.3 software (version 4.2.5; GSL Biotech; available at www.snapgene.com) aligned the annotated sequences for each gene and showed differences in sequence and translation (Figure S1). Nine genes associated with the Hd trait displayed 41 variants, of which 23 variants were non-causal variants, as they were common between the bulks but different from the reference; this type of polymorphisms is not beneficial, thus it was ignored. Of the remaining 18 variants that were polymorphic/segregating within/between the bulks, only 12 were non-synonymous and hence considered to be possible causal variants (Table 2). These 12 non-synonymous causal variants existed in six genes (*Hd3a*, *Hd3b*, *Hd5*, *Ehd1*, *Ehd2,* and *Ehd3*) on three different chromosomes (6, 8, and 10). The accuracy of the variants was examined thoroughly by checking the integrity of mapping and the frequency of each polymorphism in both the HdL and the HdH bulks. According to the frequency limits followed in the statistical analysis, only two variations, one multi nucleotide polymorphism (MNP) and one insertion (Ins) (Table 2), clearly distinguished between the two bulks, HdH and HdL. One of these variations, one MNP CC > AA, which caused an amino acid change, Pro179Asn, was found at the position 2942292^2942293 bp on chromosome 6 within the sequence of the fourth exon of the *Hd3a* gene in the HdH-bulk genome at high level of frequency (80%) and coverage (28×) (Figure S1A). Regarding the second variant, fifteen individuals (100%) in the LH-bulk showed an amino acid insertion, 277insAsn, due to insertion of three bases, TGT, at position (14740495^14740496) bp on chromosome 10 within the sequence of the third exon of the *Ehd2* gene at a high coverage of 39×. Blast2Go software showed that no KEGG pathway maps were found.

Ten key genes that control and regulate tiller number were scanned in this study. The total number of variants in these tiller-related genes was 27, of which 23 causal variants distinguished the two extreme-phenotypic bulks of the tiller trait, TL and TH. However, only 12 were nonsynonymous variants, and only two were potentially causal variants (Table 2). The tillering high-bulk contained both non-synonymous variants in two different genes, *D10* and *D53* (Figure S1B), at a high frequency level, 86% and 75%, and coverage of 49× and 57×, respectively; that means about 12 individuals out of 15 have these variations (Table 2). In contrast, the frequency for the variants in the tillering low-bulk (TL-bulk) was very low, only 11% and 12%, respectively. The variation in the sequence of *D10* gene was a SNP (T > G) that was located at the 31,225,473 bp position on chromosome 1 within the fifth exon and resulted in an amino acid substitution Lys565Asn. While the other variation was an SNP (G > A) at position 199,002 bp on chromosome 11 in the sequence of the third exon of *D53* gene, this variation caused an amino acid change (Ala1113Thr). When the annotated sequences of the *D10* and the *D53* genes were imported into Blast2GO software, the results showed one KEGG pathway, which was carotenoid biosynthesis involving the annotated sequence of *D10* gene, and an enzyme “1.13.11.69-synthase” (Figure 2B).

Five genes associated with the TGW trait displayed 14 variants, of which eight variants were ignored because they were synonymous variants. Only six variants were found between low and high bulks, TGWL and TGWH; one of them was synonymous, while five variants were non-synonymous variants (Table 2). These non-synonymous variants are located within four genes (*Gn1a*, *GS3*, *GIF1*, and *GW5*) on four different chromosomes (1, 3, 4, and 5). According to the frequency limits followed in the statistical analysis, three variants, two SNPs and one insertion, were found in two different genes, *Gn1a* and *GIF1* (Table 2; Figure S1C). Two variants, one SNP (C > A) and one Ins (CGG)*^2^, were found at the positions 5,275,210 and 5275281^5275282 bp on chromosome 1 within the first exon of *Gn1a* gene in the high bulk for the TGW trait at a high level of frequency, 88% and 77%, and coverage of 40× and 30×, respectively. In the *GIF1* gene, there was an SNP (A > G) in 84% of the individuals in the low bulk of TGW trait at the position 20,422,339 bp on the first exon of chromosome 4 with coverage of 32×. The annotated sequences of these two genes, *Gn1a,* which encoded 1.5.99.12-dehydrogenase enzyme (cytokinin dehydrogenase 2 (CKX2)), and *GIF1*, which encoded 3.2.1.26-invertase enzyme (cell-wall invertase), were involved in three KEGG pathways, cytokinin signalling pathway via zeatin biosynthesis (Figure 3B), starch and sucrose metabolism (Figure 4), and galactose metabolism, respectively.

## 3. Discussion

As already highlighted, the recent advances of NGS resulted in developments in genotyping technologies and thus all genotyping-dependent technologies such as GWAS. However, even with the advances of genotyping technologies, the accurate conventional GWAS analyses is still time-consuming, labour intensive, and expensive, as these experiments require genotyping of a large number of individuals. Therefore, the NGS-based BSA approach has been proposed to overcome this limitation. The advantage of this approach is not only the ability to minimise the number of individuals to be genotyped but also the ability to detect even loci of small effect by enriching rare alleles and boosting their effects [51].

In order to ensure maximum benefits from this approach, it is imperative to consider the factors that directly affect NGS-based BSA, including phenotyping accuracy, bulk size, and sequence depth. Yang et al. [39] tested the impact of the accuracy of phenotypic data and found that this analysis can tolerate inaccuracies in the phenotypic data to some extent, however, they proposed improving the association analysis through precise phenotyping. In this study, the phenotypic data were precisely assayed by Africa Rice Centre for many generations, and only two generations, BC2F9 (2012) and BC2F10 (2013), were considered. The phenotypic data for both generations were thoroughly studied, where individuals were selected with relatively constant values in both generations while individuals with a huge difference between the trait values in both generations were excluded; through this step, NGS-based BSA analysis is expected to have more power. In addition to the precision of phenotyping, bulk size is also critical. In our study, the pools (bulk) sizes were approximately 10% of the population (15*2 individuals*bulk out of 285 backcross inbred lines (BILs) (Figure 1). This study design was a test of the proposals of some studies. Fhrenreich et al. [51] suggested using bulk sizes higher than 5% of the phenotyped population, whereas Magwene et al. [52] suggested increased bulk sizes as large as 10% assuming adequate amounts of quantitative genotyping. Moreover, the depth of sequencing in the current study (Table S1) was higher than that recommended previously, where Magwene et al. [52] recommended sequencing the genetic materials at a depth as high as the number of individuals in a bulk to accurately estimate allele frequencies.

Our association analysis pipeline involved multiple steps of variant calling and filtration. Allele frequency was one of the most critical parameters for variant calling tools and variant filtration analysis. Consequently, NGS-based BSA would be more powerful when using adequate sequence depth. In order to call the actual SNPs and avoid false-positive signals, two solutions were applied. First through the sequencing process, during the library preparation for sequencing, the use of PCR-free libraries protocols eliminates any PCR error which can be easily considered as a true allele [53]. Secondly, through the sequence data analysis, the “InDels and Structural Variants” and the “Local Realignment” tools were applied to improve the alignment of the reads in read mapping, especially nearby insertion and deletion (InDels) positions and structural variations where the number of errors and weaknesses in read mapping is elevated [54]. Likewise, Liu et al. [55] confirmed the ability of “Local Realignment” to efficiently minimise false-positive variants depended on the depth of sequencing.

Variants were called through sequential steps, starting with variant detection where various settings were assessed. Setting 1 with the most relaxed frequency increased the number of common variants identified across the opposite bulks comparing with other sets of setting, thus avoiding the risk of missing out low frequency SNPs that might be important for analysis. Thereafter, the number of variants was reduced on the basis of being synonymous or non-synonymous variants. Finally, based on zygosity and frequency, the set of non-synonymous variants for each trait was filtered and then grouped into four categories. Interestingly, in this study, the hom-hom category was present, although it had the lowest number of SNPs among the categories (Table S3), in contrast to the report of Tran et al. [56] that no hom-hom category was observed in a similar study.

### 3.1. General Association Analysis

Functional variation associated with traits of interest may be present in any part of the genome. However, the analysis of non-synonymous SNPs in candidate genes, as used here, is a useful first analysis and has been employed in other systems [56]. The functional annotation analysis of data for the Hd trait resulted in identification of 21 KEGG pathways, among which only one pathway was linked to the Hd trait directly; this pathway was the abscisic-acid (ABA) biosynthesis (Figure 2A). This association was confirmed by Yang et al. [57] in which the early-flowering spikelets had a high ABA content in contrast to later-flowering spikelets that contained low levels of ABA. Additionally, a recent study [15] revealed that the expression of some heading date-related genes, including *Hd3a* gene, can be regulated by ABA, where the accumulation of ABA eventually leads to early heading and flowering. In this KEGG pathway, a single non-synonymous SNP from the het-het category was found in the annotated sequence of “*NCED5*” gene within HdH-bulk. The *NCED5* gene encodes OsNCED5, which catalyses the key step in abscisic-acid (ABA) biosynthesis, where the overexpression of OsNCED5 enzyme causes ABA accumulation [33]. This SNP located at the position of 3,151,977 bp on chromosome 6 as A-alleles within the HdH-bulk at a frequency of 82% and coverage of 51× while the frequency of A-allele at the same position but within the HdL-bulk was 19% with a coverage of 54× (Table 1). Therefore, this SNP, which results in a change of amino acid from leucine to phenylalanine (Leu151Phe) in the *NCED5* gene, may be one of the heading date-associated variants that repressed ABA biosynthesis and thus the ABA-dependent pathway in the HdH-bulk, causing late heading.

When the annotated sequences of tillering trait were imported into Blast2GO, the results showed that, among 97 KEGG pathways, two pathways, carotenoid biosynthesis and zeatin biosynthesis (Figure 2B and Figure 3A), were associated with tillering trait. The biosynthesis pathway of SLs is regulated by *Dwarf* genes, including *D10* gene that encodes carotenoid cleavage dioxygenase 8 homolog B, chloroplastic (OsCCD8b) (Figure 2B), whereas *D10* with other *Dwarf* genes, *D17* and *D27*, control tiller bud outgrowth, the second stage of tillering after the formation of an axillary bud [22,25,27]. In this analysis, the annotated sequence of *D10* (LOC4326177) gene, which encodes “1.13.11.69-synthase” enzyme, had SNPs positioned at 31,225,473 bp on chromosome 1 with a G-allele frequency of 86% in the TH-bulk and 11% in the TL-bulk at a coverage of 49× and 35×, respectively, resulting in a change of an amino acid from lysine to asparagine (Lys565Asn) (Table 1). This SNP may have an essential role in decreasing the level of strigolactones, the repressor of tillering.

According to Hussien et al. [9], cytokinin has an indirect negative effect on the SLs signalling pathways, particularly on the product of the *D14* gene [27]. Additionally, Dun et al. [23] demonstrated that some mutants with overexpression of cytokinin show more shoot branching. In this study, a single non-synonymous SNP was found in the annotated sequence of the LOC4334529 gene, which encodes “2.5.1.75–dimethylallyl transferase” enzyme. This SNP was positioned at 33,906,284 bp on chromosome 3 with a G-allele frequency of 14% in the TL-bulk and 96% in the TH-bulk at a coverage of 36× and 50×, respectively, resulting in a change of amino acid from serine to glycine (Ser113Gly) (Table 1). This SNP seems to play an essential role in increasing the level of cytokinin and thus tillering, consistent with the study of Sakamoto et al. [58], which found that the high content of CK was due to overexpression of *OsIPTs* genes in some OsIPTs transformants increasing the activity of the axillary bud.

For 1000 grain weight trait, the 62 KEGG pathways produced by Blast2GO were thoroughly examined to record any SNP related to this trait. There were three genes present in three pathways, zeatin biosynthesis, carotenoid biosynthesis, and starch and sucrose metabolism, which were associated with the TGW trait. In the sequence of the first gene, LOC107275425, involved in the biosynthesis of cis-type cytokinin (Figure 3A), there was a non-synonymous SNP with a higher C-allele frequency of 80% in the TGWL-bulk than in the TGWH-bulk (18%) resulting in a change of amino acid from serine to proline (Ser73Pro). The coverage at position 4,834,141 bp on chromosome 7 for this SNP was 51× and 32× in the low and the high bulks, respectively (Table 1). According to Murai [32], isopentenyl pyrophosphate transferase (IPT), which is encoded by *IPT6*, is an essential enzyme for cytokinin synthesis, and it has a positive effect on cytokinin accumulation, which modulates the number of reproductive organs and thus results in enhanced grain production. In conclusion, a non-synonymous variation in this gene within the TGWL-bulk means this variation may affect the cytokinin synthesis negatively, thus reducing the number of grains.

Furthermore, the position 1,971,432 bp on chromosome 4 in the TGWH-bulk had an A-allele instead of a G-allele (TGWL-bulk allele) that led to an amino acid change from arginine to glutamine (Arg94Gln) (Table 1). This non-synonymous variation was located in the sequence of the LOC107275432 gene that encodes “1.13.11.51-dioxygenase” enzyme. This enzyme acts as the main enzyme in ABA biosynthesis pathway (Figure 2A). As mentioned earlier, rice plants that produce large and heavy grains showed high ABA content [57], as ABA hormone is one of the key regulators of some of the genes involved in starch biosynthesis, thus ABA is positively correlated with the grain filling trait [7,50,57]. In 2006, Yang et al. [57] reported that the early-flowering spikelets which generally fill fast and produce large and heavy grains had a high ABA content in contrast to later-flowering spikelets that contain low levels of ABA. Accordingly, presenting a non-synonymous variation in the sequence of the LOC107275432 gene in 97% of the TGWH-bulk means that this variation has a positive effect on the ABA synthesis and thus starch biosynthesis. Therefore, this variation can be considered as one of the effective 1000 grain weight trait-associated variants.

Lastly, a non-synonymous SNP (A > G) was distinguished in the TGWL-bulk with a G-allele frequency of 84%, while in the TGWH-bulk, it was 18%, resulting in a change of amino acid from threonine to alanine (Thr40Ala). This SNP was positioned at 20,422,339 bp on chromosome 4 within the sequence of LOC4335790 (*GIF1*) gene with the coverage of 32× and 38× in low and high bulks, respectively (Table 1). As *GIF1* gene regulates the levels of sugar for starch synthesis during early grain-filling, this gene can be useful for increasing the size of the grain (filling) when it is expressed abundantly. Finding a non-synonymous SNP in the TGWL-bulk at a frequency of 84% indicates that this variant repressed the expression of *GIF1* gene and, in turn, led to weight reduction. Comparably, a study revealed that deletion of 1 bp in the fourth exon of the *GIF1* gene that generated a premature stop codon (TAA) slowed grain filling and thus reduced grain weight by 24% in gif1 mutants compared to wild-type rice [10].

### 3.2. Candidate Genes Analysis

In this study, the contributions of nine candidate genes to the heading date trait were thoroughly evaluated; and only two non-synonymous causal variants corresponded to the frequency limits followed in the statistical analysis. These two variations (one MNP and one Ins; (Table 2)) in the sequence of two different genes (*Hd3a* and *Ehd2*) clearly distinguished the high and the low bulk of the Hd trait (Figure S1A). According to many studies, the *Hd3a* gene is one of the key genes of flowering time within the Hd1 photoperiod pathway, as it is responsible for promoting floral transition under the control of *Hd1* gene under short day (SD) conditions [12,14,15]. Moreover, Takahashi et al. [21] revealed that the variations in the sequence of *Hd3a* along with other genes, *Hd1* and *Ehd1*, lead to heading date diversity. Therefore, the variation (one MNP CC > AA) found in the sequence of *Hd3a* that caused an amino acid change, Pro179Asn, might be responsible for the delay in heading date in the HdH-bulk.

With respect to the second variation found in the sequence of *Ehd2* gene, the high average coverage (39×) at the variant location for both bulks and the significant difference in the allele-frequency values between the two bulks, 100% in the HdL-bulk compared to 20% in the HdH-bulk (Table 2), indicate that this variation is undoubtedly associated with the early heading date trait. As already highlighted, the *Ehd2* gene along with other upregulation genes promotes the function of *Ehd1* gene within the Ehd1 photoperiod pathway, unique to rice, under unfavourable day-length conditions [20]. Therefore, this insertion of an amino acid, asparagine, within the expression product of *Ehd2* gene, caused a real functional variation that positively impacted the Ehd1 photoperiod pathway comparing to the insertion of 4 bp within the second exon of the same gene that was revealed by Matsubara et al. [19], which was an unfunctional variation resulting in a late-heading date.

Ten genes were scanned searching for trait variants associated with tillering. Similar to the Hd trait, there were only two non-synonymous causal variants meeting the requirements for verifying accuracy. Both variations were found in the TH-bulk in the sequence of two different genes, *D10* and *D53* (Figure S1B). However, the results of Blast2GO software showed one KEGG pathway, carotenoid biosynthesis, involving the annotated sequence of *D10* gene (Figure 2B). This variation, SNP (T > G) at position 31,225,473 bp, within the annotated sequence of *D10* gene was previously revealed in the general association analysis (Table 1).

In terms of D53, which acts as a repressor of SLs-signalling, thereby enhancing the axillary bud outgrowth in rice [8,22,27], there was no KEGG pathway related with the annotated sequence of *D53* gene. However, the presence of a non-synonymous causal SNP in the sequence of *D53* gene of 11 individuals from TH-bulk matched with the observation of the study of Zhou et al. [27], where a mutation in *D53* gene, an SNP and 15 bp deletion in the third exon, which was defined as a gain-of-function mutation, caused accumulation of the D53 protein, which blocked the SLs-signalling pathway and induced tiller formation and thus produced a larger number of tillers. A similar observation has been reported by Jiang et al. [8]. As the tiller number mainly influences the panicle number in rice, mutants in SL biosynthesis and signalling are extremely useful in plant breeding for high productivity [25].

The data analysis of the 1000 grain weight trait determined 14 variants within the genome of five TGW-associated genes; however, only three variants (two SNPs and one insertion) in the genome of two different genes (*Gn1a* and *GIF1*) were selected according to the frequency limits followed in the statistical analysis (Table 2 and Figure S1C). In this analysis, the annotated sequence of the *Gn1a* gene was involved in one KEGG pathway, zeatin biosynthesis (Figure 3B), because this gene plays a pivotal role in cytokinin metabolism by encoding oxidase/dehydrogenase cytokinins (CKX2) that degrades cytokinin, where the level of accumulation of cytokinin modulates the number of reproductive organs, thus enhancing grain production [31,32]. Consequently, the higher grain number might be accounted for by any variants that can cause low expression of the *Gn1a* gene and thus the CKX2 enzyme. In this study, two variants, one SNP (C > A) and one Ins (CGG)*^2^, were found in the *Gn1a* gene of 12 to 13 individuals in the TGWH bulk, while only two individuals showed these variations in the TGWL bulk. These variations may lead to a low level of CKX2 expression and thus an increase in the accumulation of CK, thus causing the difference in the weight of 1000 grain between high and low value bulks (Table S8). This finding is consistent with what has been found by Ashikari et al. [31] and reviewed by Murai [32]; a mutation that caused extremely low expression of *OsCKX2* was found in high-yielding rice varieties. In the annotated sequence for *GIF1* gene that encodes cell-wall invertase enzyme within the starch and sucrose metabolism pathway, there was an SNP (A > G) at 20,422,339 bp previously revealed in the general association analysis.

## 4. Conclusions

In this study, all variations detected had higher allele frequencies in one bulk than in the other bulk. This result was consistent with the Schlötterer et al. [59] study, which stated that the allele frequency could easily distinguish causal and non-causal variants, where the frequency of causal variants is different between bulks while the frequency of non-causal variants is the same in both bulks. Additionally, the sequence depth of positions of variation was very high in both bulks of each trait, indicating the high quality of this polymorphism, similar to that reported by Tran et al. [56]. Therefore, all the SNPs described above are likely to be significant TAVs for rice breeding; however, they should also be subjected to further functional research for validation.

## 5. Materials and Methods

### 5.1. Plant Materials and Study Design

A population of a total of 285 backcross inbred lines (BILs) was provided by Africa Rice Center (AfricaRiceat the Research Station in Bouake (Cote d’Ivoire); http://www.africarice.org). This population was developed from crossbreeding between WAB56-104 (*O. sativa ssp japonica*), which was the recurrent parent, and CG14 (*O. glaberrima* Steud.), which was the donor parent [60]. The population was phenotyped for several agronomic traits for many generations by Africa Rice Center. Traits were characterized thoroughly for two generations BC2F9 (May 2012) and BC2F10 (May 2013) (Table S9). In this study, only three quantitative agronomic traits, heading date (Hd), tiller number at maturity (T), and 1000 grain weight (TGW), were considered.

To facilitate the selection of bulks, a frequency distribution curve for each trait was plotted based on the average value of both generations (F9 and F10). Using the frequency distribution curve of the selected traits, 15 individuals (5% of the population) from each extreme phenotypic end of the curve were elected to build each bulk (Table S10, Figure 1), and two bulks are required for each trait. Therefore, only 90 individuals out of 285 were selected.

The label style of bulks was as follows: the bulks with high and low values of heading date trait are indicated as “heading date high (HdH)” and “heading date low (HdL)” respectively. Similarly, the high and the low bulks of tiller number trait are referred to as “tiller high (TH)” and “tiller low (TL)”, respectively, while high and low bulks of 1000 grain weight are referred to as “1000 grain weight high (TGWH)” and “1000 grain weight low (TGWL)”, respectively. The values of individuals in each bulk of traits are as shown in Table S8.

### 5.2. Seed Germination and Growth

About 10 seeds of each individual, a total of 900 seeds, were germinated and planted using the same protocol described by Badro et al. [61].

### 5.3. DNA Extraction, Bulk Preparation, and Sequencing

After harvesting leaves tissues, total genomic DNA was extracted individually using the CTAB (Cetyl trimethylammonium bromide) protocol described by Furtado [62] with slight modifications explained by Badro et al. [61]. The quality of DNA was assessed by NanoDrop™ 8000 Spectrophotometers (Thermo Fisher Scientific, Wilmington, DE 19810 USA) while the DNA quantity was estimated by agarose gel electrophoresis (1%, 120 V for 1 h). In order to build DNA bulks, equivalent amounts of DNA were pooled from 15 individuals for each bulk, and the DNA concentration of each bulk was then checked to adjust to 50 ng/µL, thereby, six bulks, two for each agronomic trait, were prepared for the next generation sequencing.

Six PCR-free libraries were prepared and indexed separately, then pooled together and sequenced by Illumina HiSeq 4000 flow-cell at MACROGEN (Seoul, Korea; http://dna.macrogen.com). 

### 5.4. Sequencing Data Analysis

The paired-end reads of 151 bp in size of six bulks were analysed using CLC Genomics Workbench version 11.0.1 (CLC Bio, a QIAGEN Company, Aarhus, Denmark; www.clcbio.com). The quality control (QC) tool was applied to verify the integrity of the data and to determine the appropriate trimming score. The raw sequence data were trimmed at a quality limit of “0.01”. The trimmed reads were then mapped to the reference sequence, *O. sativa* cv Nipponbare (Oryza_sativa.IRGSP-1.0 (https://plants.ensembl.org/Oryza_sativa), using default parameters except for a length fraction (LF) of 1.0 and a similarity fraction (SF) of 0.85. In order to improve the alignment of the reads in read mappings and avoid false-positive variants, the mapping file was subjected to “InDels and Structural Variants” and “Local Realignment” tools on CLC. The final output was the stand-alone mapping, known as “mapping-LR” file, and this file was used for further analysis.

### 5.5. Association Analysis

#### 5.5.1. Genotype Calling

Based on data quality (QC), three sets of variants calling parameters were implemented to determine the optimal set, in which: (1) 10, 2, and 10%, (2) 10, 2, and 20%, and (3) 10, 3, and 30% for minimum coverage, minimum count, and minimum frequency, respectively. The optimum filters for all bulks were as follows: minimum coverage of 10, read count of 2, and allele frequency of 10%. Subsequently, to identify variants between two bulks, a comparison was performed between the mapping-LR file and the variants track of the opposite bulks of each trait by using the “Identify Known Mutations from Sample Mappings” tool; in this study, this tool is referred to as the “mutation test”. Variants detected must meet the following requirements: minimum coverage of 10 and detection frequency of 10%. Ultimately, the output of the mutation test of each trait was annotated by the “Amino Acid Changes” tool to obtain just the non-synonymous variants (Figure S2A).

#### 5.5.2. Statistical Analysis of Marker-Trait Association (MTAs)

The set of non-synonymous variants was filtered based on zygosity and frequency. Four categories of comparisons were created between the two opposite bulks of each trait. These four categories were as follows: (1) hom-hom, (2) hom-het, (3) het-hom, and (4) het-het, and their frequencies were as follows: (1) >95%–>95%, (2) >95%–<30%, (3) <30%–>95%, and (4) (>70%–<30%)–(>70%–<30%), respectively. In this analysis, only variables of single nucleotide polymorphisms (SNPs) were considered. The final set of SNPs from the het-het category was subjected to further statistical analysis, where a Chi-square test was done on their allele frequency to distinguish between causative and non-causative SNPs (Figure S2A). All SNPs that had a probability (*p*-value) of < 0.01 were filtered again using the “If” command on Excel to select those that had a frequency of >80% in one bulk when the frequency in the other bulk was <20%. This extra frequency filtration ensures that the allele at a position in one bulk differs significantly from that at the same position in the opposite bulk. To verify the accuracy of the selected SNPs of all categories, a list of several tracks was created, including: the output of “Amino Acid Changes”, “Basic Variants Detection”, and “Local Realignment” tools. Additionally, all the three annotation tracks of the reference, coding sequence (CDS), transcript (mRNA), and sequence (genome) tracks, and SNPs accuracy were checked manually.

#### 5.5.3. Functional Annotation

For functional annotation analysis, a sequence of CDS that contains only the selected SNPs was extracted from a reference sequence track (annotated genome); this step was executed through the “Extract Annotations” tool on CLC. The output of extract annotations for all categories of all traits was imported into Blast2GO 5PRO software [63] separately, and the annotation process was then run under the default parameters. The general workflow of Blast2GO includes three steps: blast, mapping, and annotation, in that order. The output of this workflow can be linked to proteins, enzymes, and biosynthesis pathways through different annotation databases that support Blast2GO software. Trait-associated variants were identified after carefully inspecting all resulting enzymes and KEGG pathways.

### 5.6. Analysis of Candidate Genes

Many reviews have provided information about the genes that affect the regulation of heading date, tiller number, and 1000 grain weight traits in rice. This study involved 24 genes associated with Hd, T, and TGW traits (Table S7). To determine the variants associated with the candidate genes, the annotations of all trait-related genes were extracted using CLC genomics Workbench through the following pipeline: (i) at a low coverage threshold of 5, the consensus sequences of all chromosomes were extracted from the mapping-LR file of each bulk using the “Extract the Consensus Sequence” tool, (ii) the output was converted into three tracks, genome, genes, and CDS, utilizing the “Convert to tracks” tool, thus this step provided a database for the process of extracting annotations, (iii) the CDS tracks were separately filtered by name to select only the desired genes, finally, (iv) by using the “Extract Annotations” tool, annotations were extracted for all genes from a genome track (Figure S2B). Trait-associated variants were then detected manually by aligning the annotated sequences of each gene from the two opposite bulks and the reference with the aid of SnapGene software (version 4.2.5; GSL Biotech; available at www.snapgene.com), thoroughly investigating all variations and their translation afterwards. Finally, the accuracy of the selected TAVs was verified using the verification process (statistical analysis). After detecting TAVs and checking their accuracy, the annotated sequences of each gene were introduced to Blast2GOsoftware for more information on their functional annotation (Figure S2B).

## Figures and Tables

**Table 1:** Details of single nucleotide polymorphisms (SNPs) tightly associated with *Hd, T, and TGW traits detected by functional-annotation analysis.

*Chr/Reg	*Ref.	Allele	Low Bulk			High Bulk			*AA Change	Trait	Sequence (Gene)	Enzyme
			*Cov.	*Freq.	*Zygo.	*Cov.	*Freq.	*Zygo.				
Chr06/3151977	G	A	54	19	Het	51	82	Het	Leu151Phe	Heading (Hd)	LOC107275952	1.13.11.51-dioxygenase
Chr04/1971432	G	A	28	29	Het	28	97	Hom	Arg94Gln	1000 grain Weight (TGW)	LOC107275432	
Chr04/20422339	A	G	32	84	Het	38	18	Het	Thr40Ala		LOC4335790	3.2.1.26-invertase
Chr07/4834141	T	C	51	80	Het	38	18	Het	Ser73Pro		LOC107275425	2.5.1.75–transferase
Chr03/33906284	A	G	36	14	Het	50	96	Hom	Ser113Gly	Tillering (T)	LOC4334529	
Chr01/31225473	T	G	35	11	Het	49	86	Het	Lys565Asn		LOC4326177	1.13.11.69-synthase

*Hd: heading date, T: tiller number at maturity, TGW: 1000 grain weight, Chr/Reg: chromosome/region, Ref: reference, Cov: coverage, Freq: frequency, Zygo: zygosity, AA Change: amino acid change.

**Table 2:** Summary of the analysis of investigation of candidate genes for Hd, T, and TGW traits.

**Gene**	**Gene ID**	**Chr #**	**Length (bp)**	**Total No. Variants**	**NS-Variant**	**TAVs**	**Frequency%**		**Nuc. Change**	**aa Change**	**Bulk**
							**HdL**	**HdH**			
***DTH2***	LOC4330574	2	1224	4	0	0	-	-	-	-	-
***Hd16***	LOC4334396	3	2124	0	0	0	-	-	-	-	-
***Hd1***	LOC4340746	6	1188	9	0	0	-	-	-	-	-
***Hd3a***	LOC4340185	6	540	7	3	1	17	65	G**C**A > G**G**A	Ala100**Gly**	HdH
							15	46	**G**TC > **A**TC	Val177**Ile**	
							^†^12	80	**CC**C > **AA**C	Pro179**Asn**	
***Hd3b***	LOC4340184	6	537	5	4	0	23	50	**G**GC > **C**GC	Gly3**Arg**	HdH
							21	50	A**G**C > A**T**C	Ser4**Ile**	
							2	62	G**T**C > G**C**C	Val31**Ala**	
							0	55	A**A**G > A**C**G	Lys45**Thr**	
***Hd5***	LOC4344784	8	894	3	1	0	8	56	G**T**A > G**G**A	Tyr189**Ser**	HdH
***Ehd3***	LOC4344443	8	1692	5	1	0	62	40	Ins **CCG**	19ins **Pro**	HdL
***Ehd1***	LOC107276289	10	1026	7	2	0	**79**	**43**	TT**C** > TT**T**	Glu311**Lys**	HdL
							**78**	**44**	GT**C** > GT**T**	Asp195**Asn**	
***Ehd2***	LOC4348644	10	1428	1	1	1	^†^100	21	Ins **TGT**	77ins **Asn**	HdL
**Total**				41	12	2					
**Gene**	**Gene ID**	**Chr #**	**Length (bp)**	**Total no. variants**	**NS-Variant**	**TAVs**	**Frequency%**		**Nuc. Change**	**aa Change**	**Bulk**
							**TL**	**TH**			
***D10***	LOC4326177	1	1710	2	1	1	^†^11	86	**T**TT > **G**TT	Lys565**Asn**	TH
***RCN1***	LOC4332449	3	2364	2	0	0	-	-	-	-	-
***HTD2 (D14)***	LOC4331983	3	957	0	0	0	-	-	-	-	-
***OsTB1***	LOC4333856	3	1167	1	1	0	0	23	Ins (**GCG**)*^5^	112-1116ins Ala*^5^	TH
***HTD1 (D17)***	LOC4336591	4	1830	5	2	0	0	51	Del (**GCC**)*^4^	Ala*^4^ 41-44del	TH
							12	53	**C**TC>**G**TC	Leu334**Val**	
***DWARF3***	LOC4339885	6	1521	1	1	0	23	54	**A**CC>**T**CC	Thr500**Ser**	TH
***D3***	LOC9272469	6	2163	5	4	0	26	71	Ins (**GAG**)*^3^	7-10ins Glu*^3^	TH
							0	55	Ins **GCG**	111ins **Gly**	
							42	61	**G**GT>**T**GT	Gly498**Cys**	
							33	68	GA**T** > GA**G**	Asp514**Glu**	
***MOC1***	LOC107278653	6	1326	2	0	0	-	-	**-**	**-**	**-**
***OsSPL14***	LOC4345998	8	1257	4	1	0	51	38	**C**GG > **T**GG	Ala317**Thr**	TL
***D53***	LOC4349543	11	3396	5	2	1	8	60	Del **GAG**	**Glu** 1070del	TH
							^†^12	75	**G**CA > **A**CA	Ala1113**Thr**	
**Total**				**27**	**12**	**2**					
**Gene**	**Gene ID**	**Chr #**	**Length (bp)**	**Total No. Variants**	**NS-Variant**	**TAVs**	**Frequency%**		**Nuc. Change**	**aa Change**	**Bulk**
							**TGWL**	**TGWH**			
***Gn1a***	LOC4327333	1	1698	5	2	2	^†^14	77	**C**GC > **A**GC	Ale105**Ser**	TGWH
							^†^13	88	Ins (**CGG**)*^2^	81-82insAla*^2^	
***GW2***	LOC4328856	2	1278	1	0	0	-	-	-	-	-
***GS3***	LOC9269602	3	699	2	1	0	69	5	Del **AGG**	**Ser**123del	TGWL
***GIF1***	LOC4335790	4	1797	3	1	1	^†^84	18	**A**CC > **G**CC	Thr40**Ala**	TGWL
***GW5***	LOC4338011	5	1413	3	1	0	18	47	Del **GCG**	**Gly** 365del	TGWH
**Total**				**14**	**5**	**3**					

This summary is represented by gene, gene ID, chromosome (Chr), gene length in base pair (bp), protein length in amino acid (aa), total number of variants, variants between bulks, non-synonymous variants, trait-associated variants (TAVs), frequency of variants in low (L) and high (H) bulks, nucleotide change, amino acid change, and bulks which include TAVs (HdL: heading date low, HdH: heading date high, TL: tiller low, TH: tiller high, TGWL: 1000 grain weight low, and TGWH: 1000 grain weight high) for Hd: heading date, T: tiller number at maturity, TGW: 1000 grain weight traits; ^†^: TAVs met the requirements for verifying accuracy; DTH: days to heading, Hd: heading date, Ehd: early heading date, D: dwarf, RCN: reduce culm number, HTD: HIGH-TILLERING DWARF, OsTB: TEOSINTE BRANCHED, MOC: monoculm, Gn: grain productivity, GW: grain width, GS: grain size/shape, GIF: grain incomplete filling.

## Supplementary Materials

The following are available online at https://www.mdpi.com/2223-7747/9/12/1653/s1, Supplementary file 1: List of figures; Figure S1: Alignment of annotated sequences of CDS carrying TAVs in (A) Hd, (B) T, and (C) TGW. Figure S2: Illustration of (A) association analysis, and (B) investigation of candidate genes. Supplementary file 2: List of tables; Table S1: Summary of the output of sequencing, trimming and mapping processes for each bulk. Table S2: Detailed information of the variants generated by the genotype calling for each bulk. Table S3: Summary of the output of the statistical analysis for each trait. Table S4: Pathways, enzymes, and sequences (genes) recorded by functional-annotation analysis of 298 TAVs for Hd trait. Table S5: Pathways, enzymes, and sequences (genes) recorded by functional-annotation analysis of 3375 TAVs for T trait. Table S6: Pathways, enzymes, and sequences (genes) recorded by functional-annotation analysis of 1479 TAVs for TGW trait. Table S7: Candidate genes for the three agronomic traits, heading date, tiller number, and 1000 grain weight. Table S8: Summary of the value of each agronomic trait and its bulks. Table S9: Phenotypic data of BC2F9 and BC2F10 populations of total of 285 backcross inbred lines (BILs). Table S10: Individuals of low and high-value of Hd, T, and TGW traits used in NGS-based BSA.
