# Supplementary material for: Sequence Variants Linked to Key Traits in Interspecific Crosses between African and Asian Rice
Source: Plants (Basel). 2020 Nov 26;9(12):1653. doi: 10.3390/plants9121653 (PMC7761468; doi:10.3390/plants9121653)
Supplement: Supplementary file 1 [file plants-09-01653-s001.pdf]

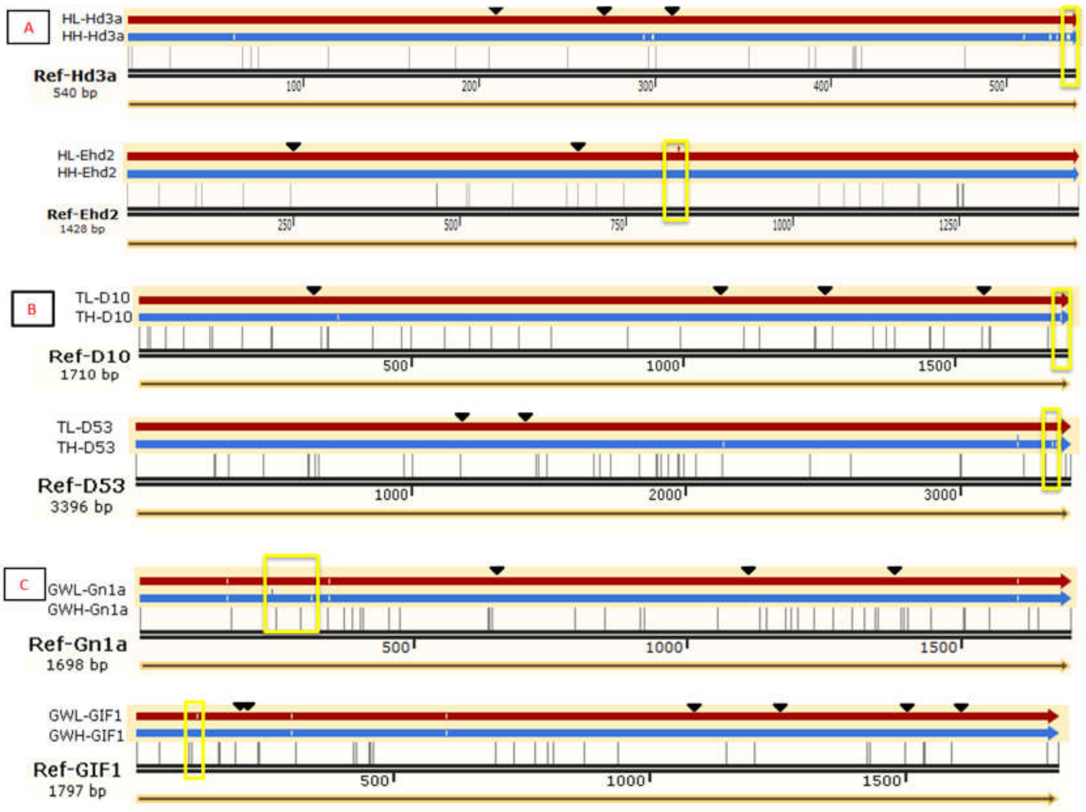

Figure S1. Alignment of annotated sequences of CDS carrying TAVs in **A**) Hd, **B**) T, and **C**) TGW.

Alignment of annotated sequences of the same gene from the Low (L, red arrows) and High (H, blue arrows) bulks to the reference (black arrows) for all CDS carrying the TAVs (yellow boxes) in **A**) Heading date (Hd) (*Hd3a* and *Ehd2*), **B**) Tiller number (T) (*D10* and *D53*), and **C**) 1000-grain weight (TGW) (*Gn1a* and *GIF1*); SnapGene 4.0.3 Software; the black arrows refer to introns' positions.

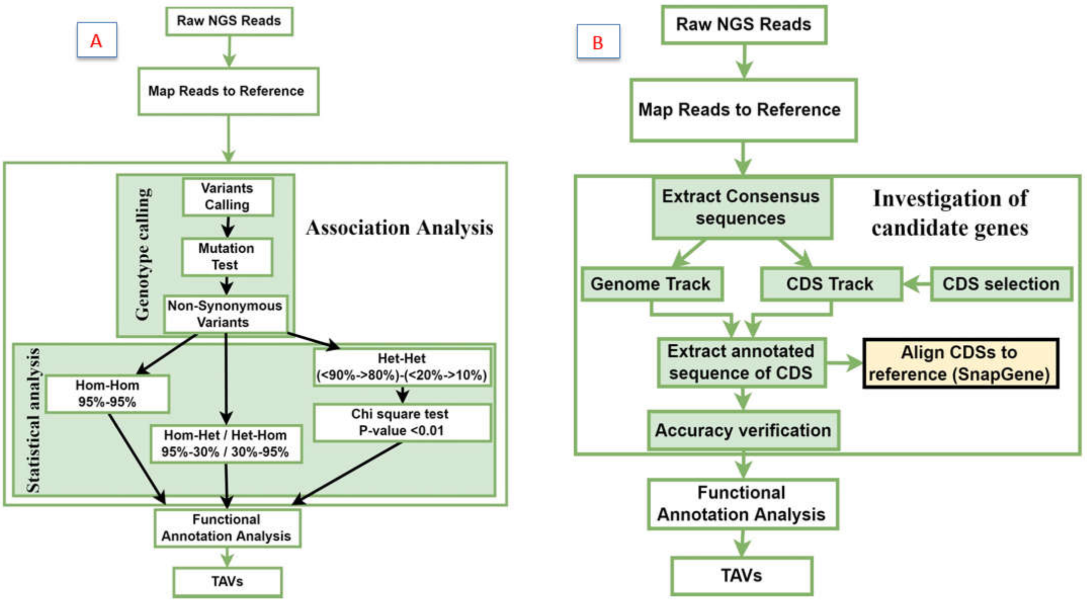

Figure S2: Illustration of A) association analysis, and B) investigation of candidate genes.

Table S1. Summary of the output of sequencing, trimming and mapping processes for each bulk.

Bulks	Raw Data					After Trimming at 0.01				After Mapping with [1]LF 1.0 and SF 0.85			
	Total Number of Nucleotides	No. of reads	Read %	Avg. Length bp	Coverage ×	No. of Reads	Read %	Avg. Length bp	Coverage ×	No. of Reads	Read %	Avg. Length bp	Coverage ×
**HdL**	25,278,846,580	167,409,580	100	151	68	149,597,483	89	125	50	123,979,357	83	125	42
**HdH**	27,256,181,312	180,504,512	100	151	73	159,017,759	88	125	53	129,111,940	81	125	44
**TL**	27,856,613,484	184,480,884	100	151	75	162,621,016	88	125	54	133,898,868	82	125	45
**TH**	33,089,827,732	219,137,932	100	151	89	199,348,728	91	126	67	159,990,164	80	126	54
**TGWL**	26,336,224,382	174,412,082	100	151	65	154,252,589	88	125	52	122,266,059	79	125	41
**TGWH**	25,930,452,048	171,724,848	100	151	69	156,074,410	91	126	53	128,762,322	83	126	44

The output is represented by the total number of reads (No. of reads), Percentage of reads (Reads %), average read length (Avg. Length bp) and average read coverage across the genome (coverage ×), where 'HdL and HdH', 'TL and TH' and 'TGWL and TGWH' refer to the low and high bulks of heading date (Hd), tiller number at maturity (T) and 1000-grain weight (TGW) traits, respectively. [1]LF: length fraction, and SF**:** similarity fraction

Table S2. Detailed information of the variants generated by the genotype calling for each bulk.

Bulks	*BVD 10,2,10%		*IKMfSM 10,10%		*AAC Non-Syn	
	Variants	SNPs	Variants	SNPs	Variants	SNPs
**HdL**	8,365,429	7,152,400	8,365,429	7,152,400	111,094	90,979
**HdH**	14,384,252	12,280,145	14,384,252	12,280,145	190,840	157,506
**TL**	8,811,568	7,504,262	8,811,568	7,504,262	119,346	97,281
**TH**	20,332,281	17,210,089	20,332,281	17,210,089	253,945	205,484
**TGWL**	19,172,478	16,282,494	19,172,478	16,282,494	246,958	201,997
**TGWH**	13,368,595	11,384,028	13,368,595	11,384,028	178,205	146,017

HdL and HdH: low and high bulks of heading date (Hd), **TL and TH:** low and high bulks of tiller number at maturity (T), **TGWL and TGWH:** low and high bulks of 1000-grain weight (TGW) traits, ***BVD:** Basic variant detection**, IKMFSM:** Identify Known Mutations from Sample Mappings**, AAC:** Amino Acid Changes; **SNPs:** Single Nucleotide Polymorphism.

Table S3. Summary of the output of the statistical analysis for each trait.

Bulks	*Hom-Hom (> 95 – > 95)				Hom-Het (>95 – < 30)			*Het-Hom (<30 – > 95)			Het-Het				Total	
											> 70 - < 30		> 80 – < 20			
	V	SNPs	SNPs after MC	Gene	V	SNPs	Gene	V	SNPs	Gene	SNPs	SNPs after Chi^2	SNPs	Gene	SNPs	Gene
**HdL-HdH**	438	127	8	5	67	40	33	89	70	56	70788	9357	180	152	298	244
**TL-TH**	2683	2150	254	213	1187	950	572	302	247	206	80928	36754	1924	1408	3375	2277
**TGWL-TGWH**	163	163	7	6	164	140	123	319	247	147	135070	51692	1085	813	1479	1043
**Total**			269	224		1130	728		564	409			3189	2373	5152	3564

The output is represented by the number of all types of variants (V), Single Nucleotide Polymorphism (SNPs), number of SNPs after manual check (SNPs after MC), number of genes (Gene) and number of SNPs after Chi-square test (SNPs after Ch^2) in all four zygosity categories (Hom-Hom, Hom-Het, Het-Hom and Het-Het), where 'HdL and HdH', 'TL and TH' and 'TGWL and TGWH' refer to the low and high bulks of heading date (Hd), tiller number at maturity (T) and 1000-grain weight (TGW) traits, respectively; ***Hom:** homozygous, and **Het:** heterozygous**.**

Table S4. Pathways, enzymes and sequences (genes) recorded by functional-annotation analysis of 298 TAVs for Hd trait.

No	Pathway	Pathway ID	#Enzs in Pathway	Enzyme	#Seqs of Enzyme	Seqs
1	Biosynthesis of antibiotics	map01130	3	ec:1.17.1.8 - reductase, ec:4.2.1.3 - hydratase, ec:2.2.1.7 - synthase	3	LOC9271805, LOC4331547, LOC4340090
2	Purine metabolism	map00230	1	ec:3.6.1.3 - adenylpyrophosphatase	3	LOC107281927, LOC4352332, LOC107276159
3	Pantothenate and CoA biosynthesis	map00770	2	ec:3.6.1.9 - diphosphatase, ec:2.7.1.24 - kinase	2	LOC4340074, LOC4331390
4	Thiamine metabolism	map00730	2	ec:3.6.1.15 - phosphatase, ec:2.2.1.7 - synthase	2	LOC107281927, LOC4340090
5	Cysteine and methionine metabolism	map00270	1	ec:2.1.1.37 - (cytosine-5-)-methyltransferase	1	LOC4331357
6	Nicotinate and nicotinamide metabolism	map00760	1	ec:3.6.1.9 - diphosphatase	1	LOC4340074
7	Folate biosynthesis	map00790	1	ec:6.3.2.17 - synthase	1	LOC4331360
8	Monobactam biosynthesis	map00261	1	ec:1.17.1.8 - reductase	1	LOC9271805
9	Carbon fixation pathways in prokaryotes	map00720	1	ec:4.2.1.3 - hydratase	1	LOC4331547
10	Carotenoid biosynthesis	map00906	1	ec:1.13.11.51 - dioxygenase	1	LOC107275952
11	Glycerolipid metabolism	map00561	1	ec:3.1.1.23 - lipase	1	LOC9270328
12	Starch and sucrose metabolism	map00500	1	ec:3.6.1.9 - diphosphatase	1	LOC4340074
13	Drug metabolism - other enzymes	map00983	1	ec:3.1.1.1 - ali-esterase	1	LOC9270328

14	Th1 and Th2 cell differentiation	map04658	1	ec:3.1.3.16 - phosphatase	1	LOC4340447
15	T cell receptor signaling pathway	map04660	1	ec:3.1.3.16 - phosphatase	1	LOC4340447
16	Amino sugar and nucleotide sugar metabolism	map00520	1	ec:2.7.1.52 - fucokinase (phosphorylating)	1	LOC4331392
17	Ether lipid metabolism	map00565	1	ec:3.1.4.4 - D	1	LOC4331421
18	Pyrimidine metabolism	map00240	1	ec:3.6.1.9 - diphosphatase	1	LOC4340074
19	Other glycan degradation	map00511	1	ec:3.2.1.51 - alpha-fucosidase	1	LOC4340204
20	Fructose and mannose metabolism	map00051	1	ec:2.7.1.52 - fucokinase (phosphorylating)	1	LOC4331392
21	Citrate cycle (TCA cycle)	map00020	1	ec:4.2.1.3 - hydratase	1	LOC4331547
			**25**	**16 unique enzymes**	**27**	**16 unique genes**

Table S5. Pathways, enzymes and sequences (genes) recorded by functional-annotation analysis of 3375 TAVs for T trait.

No	Pathway	Pathway ID	#Enzs in Pathway	Enzyme	#Seqs of Enzyme	Seqs
1	Purine metabolism	map00230	14	ec:3.6.1.15 - phosphatase, ec:2.7.7.7 - DNA polymerase, ec:2.7.4.3 - kinase, ec:2.7.6.5 - diphosphokinase, ec:3.6.1.3 - adenylpyrophosphatase, ec:6.3.4.4 - synthase, ec:6.3.4.13 - ligase, ec:2.7.7.4 - adenylyltransferase, ec:6.3.3.1 - cyclo-ligase, ec:3.6.1.13 - diphosphatase, ec:2.7.1.25 - kinase, ec:3.5.2.5 - ec:3.5.2.5 allantoinase, ec:2.7.7.6 - RNA polymerase, ec:3.5.3.9 - deiminase	146	LOC107280855, LOC4348734, LOC107281820, LOC4346514, LOC107280211, LOC4332316, LOC4334619, LOC4330659, LOC4326261, LOC107279197, LOC9269572, LOC9266862, LOC4330371, LOC9269978, LOC4337574, LOC107276769, LOC107278308, LOC4343011, LOC4348547, LOC4348944, LOC4329993, LOC4343412, LOC4330819, LOC4326410, LOC4327730, LOC4325287, LOC9267360, LOC4332326, LOC4332601, LOC4328702, LOC4326095, LOC4330426, LOC4330142, LOC4330342, LOC4350530, LOC4334150, LOC4334272, LOC4345008, LOC9266439, LOC107281927, LOC4325639, LOC4341965, LOC4352060, LOC4343066, LOC4344750, LOC4329364, LOC9271500, LOC4324268, LOC4348797, LOC4328756, LOC9266280, LOC4332853, LOC4328758, LOC4327702, LOC4337429, LOC4337825, LOC107276580, LOC4341280, LOC4336810, LOC9267698, LOC4337027, LOC4350322, LOC4341457, LOC4341778, LOC4338040, LOC4348805, LOC4345533, LOC4352456, LOC4352733, LOC4333877, LOC4333998, LOC4350436, LOC107279989, LOC107277526, LOC107276397, LOC9269802, LOC4338568, LOC4335014, LOC4336785, LOC9271500, LOC4325305, LOC4344791, LOC4343011, LOC9270381, LOC4348734, LOC4346514, LOC4330659, LOC4326261, LOC9269572, LOC4337574, LOC107278308, LOC4343011, LOC4329993, LOC4343412, LOC4326410, LOC4327730, LOC4325287, LOC4332326, LOC4332601, LOC4328702, LOC4330426, LOC4330142, LOC4330342, LOC4350530, LOC4334150, LOC4334272, LOC107281927, LOC4325639, LOC4341965, LOC4352060, LOC4344750, LOC4324268, LOC4348797, LOC9266280, LOC4332853, LOC4328758, LOC4327702, LOC4337429, LOC4337825, LOC107276580, LOC4341280, LOC4336810, LOC9267698, LOC4337027, LOC4341778, LOC4338040, LOC4352456,

						LOC4333877, LOC4333998, LOC4350436, LOC107276397, LOC9269802, LOC4338568, LOC4335014, LOC4331792, LOC4334671, LOC4344854, LOC4334932, LOC4334671, LOC4346295, LOC4334932, LOC9272308, LOC4337429, LOC4343132, LOC9268125, LOC4328330, LOC107277526, LOC107279949
2	Thiamine metabolis m	m a p 0 0 7 3 0	3	ec:3.6.1.15 - phosphatase, ec:2.7.4.3 - kinase, ec:3.1.3.2 - phosphatase	87	LOC107280855, LOC4348734, LOC107281820, LOC4346514, LOC107280211, LOC4332316, LOC4334619, LOC4330659, LOC4326261, LOC107279197, LOC9269572, LOC9266862, LOC4330371, LOC9269978, LOC4337574, LOC107276769, LOC107278308, LOC4343011, LOC4348547, LOC4348944, LOC4329993, LOC4343412, LOC4330819, LOC4326410, LOC4327730, LOC4325287, LOC9267360, LOC4332326, LOC4332601, LOC4328702, LOC4326095, LOC4330426, LOC4330142, LOC4330342, LOC4350530, LOC4334150, LOC4334272, LOC4345008, LOC9266439, LOC107281927, LOC4325639, LOC4341965, LOC4352060, LOC4343066, LOC4344750, LOC4329364, LOC9271500, LOC4324268, LOC4348797, LOC4328756, LOC9266280, LOC4332853, LOC4328758, LOC4327702, LOC4337429, LOC4337825, LOC107276580, LOC4341280, LOC4336810, LOC9267698, LOC4337027, LOC4350322, LOC4341457, LOC4341778, LOC4338040, LOC4348805, LOC4345533, LOC4352456, LOC4352733, LOC4333877, LOC4333998, LOC4350436, LOC107279989, LOC107277526, LOC107276397, LOC9269802, LOC4338568, LOC4335014, LOC4336785, LOC4343011, LOC4332737, LOC9269151, LOC4338023, LOC4334778, LOC4338021, LOC9270081, LOC4327297
3	Biosynthe sis of antibiotics	m a p 0 1 1 3 0	33	ec:2.7.4.2 - kinase, ec:1.3.5.1 - dehydrogenase, ec:4.1.1.32 - carboxykinase (GTP), ec:5.1.3.13 - 3,5-epimerase, ec:5.3.1.9 - isomerase, ec:2.3.1.12 - acetyltransferase, ec:1.4.1.14 - synthase (NADH), ec:2.7.4.3 - kinase, ec:1.1.1.25 - dehydrogenase, ec:2.3.3.8 - citrate synthase, ec:2.7.7.64 - uridylyltransferase, ec:2.7.2.3 - kinase, ec:4.2.1.10 - dehydratase, ec:4.3.2.1 - lyase, ec:3.5.1.16 - deacetylase, ec:1.1.1.282 - dehydrogenase, ec:2.7.1.1 - hexokinase type IV glucokinase, ec:2.7.7.9 - uridylyltransferase, ec:6.3.4.13 - ligase, ec:4.2.1.20 - synthase, ec:4.3.1.19 - ammonia-lyase, ec:2.7.7.4 - adenylyltransferase, ec:1.1.1.37 - dehydrogenase, ec:4.6.1.12 - 2,4-cyclodiphosphate synthase, ec:3.1.3.3 - phosphatase, ec:2.7.1.2 - glucokinase (phosphorylating), ec:6.3.3.1 - cyclo-ligase, ec:5.4.2.8 - mannose phosphomutase, ec:4.1.1.20 -	39	LOC4332275, LOC4343404, LOC4346699, LOC9267465, LOC4340677, LOC4343003, LOC4324398, LOC4343011, LOC4325444, LOC9272295, LOC4328091, LOC4326201, LOC4348728, LOC4325444, LOC107275838, LOC4330380, LOC4325444, LOC107275630, LOC4324666, LOC4328091, LOC4326201, LOC4334671, LOC4344854, LOC4334425, LOC4333898, LOC4334932, LOC4327423, LOC4330320, LOC4330100, LOC107275630, LOC4324666, LOC4334671, LOC4341038, LOC4329234, LOC4323939, LOC4327196, LOC4338034, LOC4331917, LOC9267465

				decarboxylase, ec:2.5.1.29 - diphosphate synthase, ec:2.7.1.11 - phosphohexokinase, ec:5.5.1.4 - synthase, ec:1.1.1.133 - reductase		
4	Amino sugar and nucleotide sugar metabolism	map00520	15	ec:5.3.1.9 - isomerase, ec:3.2.1.55 - end alpha-L-arabinofuranosidase, ec:2.7.7.64 - uridylyltransferase, ec:2.4.1.43 - 4-alpha-galacturonosyltransferase, ec:3.2.1.37 - 1,4-beta-xylosidase, ec:2.7.1.1 - hexokinase type IV glucokinase, ec:2.7.7.9 - uridylyltransferase, ec:2.7.1.7 - mannokinase (phosphorylating), ec:4.1.1.35 - decarboxylase, ec:2.7.1.2 - glucokinase (phosphorylating), ec:1.1.1.22 - 6-dehydrogenase, ec:5.4.2.8 - mannose phosphomutase, ec:3.2.1.14 - ChiC, ec:2.7.7.13 - guanylyltransferase, ec:2.7.1.4 - fructokinase (phosphorylating)	33	LOC4340677, LOC4341522, LOC4328608, LOC4341521, LOC107280359, LOC4326722, LOC4351402, LOC4328091, LOC4326201, LOC4332034, LOC4326722, LOC107275630, LOC4324666, LOC4328091, LOC4326201, LOC107275630, LOC4324666, LOC4327295, LOC107275630, LOC4324666, LOC4333410, LOC4341038, LOC4327951, LOC4351195, LOC4323899, LOC4338209, LOC107281711, LOC4348631, LOC4350476, LOC4338720, LOC4332373, LOC107275630, LOC4324666
5	Aminobenzoate degradation	map00627	1	ec:3.1.3.41 - nitrophenyl phosphatase	27	LOC107275989, LOC4334153, LOC4332194, LOC4329322, LOC4329882, LOC4332737, LOC9269151, LOC4334778, LOC4334038, LOC9270081, LOC4350524, LOC4335297, LOC4338023, LOC4338021, LOC4328780, LOC4329650, LOC4324753, LOC4346981, LOC4327297, LOC4348468, LOC4326496, LOC107277360, LOC4333499, LOC4351304, LOC4330100, LOC4351686, LOC4351685
6	Starch and sucrose metabolism	map00500	15	ec:3.2.1.4 - endo-1,4-beta-D-glucanase, ec:5.3.1.9 - isomerase, ec:2.4.1.13 - synthase, ec:3.2.1.26 - invertase, ec:2.4.1.14 - synthase, ec:3.2.1.20 - maltase, ec:2.7.1.1 - hexokinase type IV glucokinase, ec:2.7.7.9 - uridylyltransferase, ec:2.4.1.15 - synthase (UDP-forming), ec:3.2.1.21 - gentiobiase, ec:2.7.1.2 - glucokinase (phosphorylating), ec:3.1.3.12 - trehalose 6-phosphatase, ec:3.2.1.2 - saccharogen amylase, ec:2.4.1.12 - synthase (UDP-forming), ec:2.7.1.4 - fructokinase (phosphorylating)	27	LOC4327286, LOC4337698, LOC4340677, LOC4341642, LOC4329561, LOC4341642, LOC4332068, LOC107275630, LOC4324666, LOC4328091, LOC4326201, LOC4346981, LOC9270758, LOC4338560, LOC107275630, LOC4324666, LOC4346981, LOC4343510, LOC9268236, LOC4343551, LOC107281242, LOC9266650, LOC4343554, LOC4343555, LOC4343552, LOC107275630, LOC4324666
7	Phenylpropanoid biosynthesis	map00940	4	ec:1.1.1.195 - dehydrogenase, ec:3.2.1.21 - gentiobiase, ec:1.2.1.44 - reductase, ec:1.11.1.7 - lactoperoxidase	20	LOC9269515, LOC4348690, LOC9270758, LOC4338560, LOC4346551, LOC4337892, LOC107281419, LOC4326968, LOC4327001, LOC4326027, LOC4347520, LOC4328842, LOC4326273, LOC4337726, LOC4337727, LOC4337725, LOC4332175, LOC9269736, LOC4332782, LOC4351300

8	Drug metabolism - other enzymes	map00983	4	ec:3.1.1.1 - ali-esterase, ec:3.2.1.31 - beta-glucuronide glucuronohydrolase glucuronidase, ec:2.5.1.18 - transferase, ec:2.3.1.5 - N-acetyltransferase	18	LOC4338583, LOC4327832, LOC107276812, LOC4338538, LOC9270328, LOC4348648, LOC107275275, LOC4330443, LOC4326787, LOC107277255, LOC4332033, LOC4348461, LOC4348549, LOC4334126, LOC4347190, LOC107276987, LOC4341999, LOC4344443
9	Inositol phosphate metabolism	map00562	10	ec:2.7.1.67 - 4-kinase, ec:3.1.4.11 - phospholipase C, ec:2.7.1.159 - 5/6-kinase, ec:3.1.3.36 - 5-phosphatase, ec:2.7.1.68 - 5-kinase, ec:2.7.1.134 - 1-kinase, ec:3.1.3.56 - 5-phosphatase, ec:3.1.4.3 - C, ec:2.7.1.150 - 5-kinase, ec:5.5.1.4 - synthase	14	LOC4333883, LOC4329953, LOC4329322, LOC4332146, LOC4332194, LOC4333499, LOC9267438, LOC4331188, LOC4329322, LOC4332146, LOC4333499, LOC4329953, LOC4340685, LOC4331917
10	Pentose and glucuronate interconversions	map00040	9	ec:3.2.1.15 - pectin depolymerase, ec:2.7.1.47 - D-ribulokinase (phosphorylating), ec:4.2.2.2 - lyase, ec:2.7.7.64 - uridylyltransferase, ec:2.7.7.9 - uridylyltransferase, ec:3.2.1.31 - beta-glucuronide glucuronohydrolase glucuronidase, ec:3.2.1.67 - 1,4-alpha-galacturonidase, ec:1.1.1.22 - 6-dehydrogenase, ec:3.1.1.11 - pectin demethoxylase	14	LOC4330924, LOC4341243, LOC4326618, LOC4352486, LOC9267504, LOC107280660, LOC4328091, LOC4326201, LOC4328091, LOC4326201, LOC4332033, LOC4341243, LOC4333410, LOC4338538
11	Galactose metabolism	map00052	8	ec:3.2.1.26 - invertase, ec:2.7.7.64 - uridylyltransferase, ec:2.4.1.82 - galactosyltransferase, ec:3.2.1.20 - maltase, ec:2.7.1.1 - hexokinase type IV glucokinase, ec:2.7.7.9 - uridylyltransferase, ec:2.7.1.2 - glucokinase (phosphorylating), ec:2.7.1.11 - phosphohexokinase	13	LOC4329561, LOC4328091, LOC4326201, LOC4325200, LOC4332068, LOC107275630, LOC4324666, LOC4328091, LOC4326201, LOC107275630, LOC4324666, LOC4327196, LOC4338034
12	Glycolysis / Gluconeogenesis	map0	7	ec:4.1.1.32 - carboxykinase (GTP), ec:5.3.1.9 - isomerase, ec:2.3.1.12 - acetyltransferase, ec:2.7.2.3 - kinase, ec:2.7.1.1 - hexokinase type IV glucokinase, ec:2.7.1.2 - glucokinase (phosphorylating), ec:2.7.1.11 - phosphohexokinase	10	LOC4346699, LOC4340677, LOC4343003, LOC4348728, LOC107275630, LOC4324666, LOC107275630, LOC4324666, LOC4327196, LOC4338034

		0010				
13	Aminoacyl-tRNA biosynthesis	map00970	6	ec:6.1.1.18 - ligase, ec:6.1.1.2 - ligase, ec:6.1.1.10 - ligase, ec:6.1.1.17 - ligase, ec:6.1.1.6 - ligase, ec:6.1.1.1 - ligase	10	LOC4325653, LOC4325167, LOC4348464, LOC4344859, LOC4332018, LOC4348562, LOC4325167, LOC4348464, LOC4330025, LOC4344859
14	Phosphatidylinositol signaling system	map04070	7	ec:2.7.1.67 - 4-kinase, ec:3.1.4.11 - phospholipase C, ec:2.7.1.159 - 5/6-kinase, ec:3.1.3.36 - 5-phosphatase, ec:2.7.1.68 - 5-kinase, ec:3.1.3.56 - 5-phosphatase, ec:2.7.1.150 - 5-kinase	10	LOC4333883, LOC4329953, LOC4329322, LOC4332146, LOC4332194, LOC4333499, LOC9267438, LOC4331188, LOC4333499, LOC4340685
15	Pyrimidine metabolism	map00240	5	ec:6.3.5.5 - synthase (glutamine-hydrolysing), ec:2.7.7.7 - DNA polymerase, ec:2.1.1.45 - synthase, ec:2.7.1.83 - kinase, ec:2.7.7.6 - RNA polymerase	10	LOC4330474, LOC9271500, LOC4325305, LOC4344791, LOC4350527, LOC4338003, LOC4343132, LOC9268125, LOC4328330, LOC107277526
16	Fructose and mannose metabolism	map00051	6	ec:2.7.1.1 - hexokinase type IV glucokinase, ec:2.7.1.7 - mannokinase (phosphorylating), ec:5.4.2.8 - mannose phosphomutase, ec:2.7.7.13 - guanylyltransferase, ec:2.7.1.11 - phosphohexokinase, ec:2.7.1.4 - fructokinase (phosphorylating)	10	LOC107275630, LOC4324666, LOC107275630, LOC4324666, LOC4341038, LOC4332373, LOC4327196, LOC4338034, LOC107275630, LOC4324666

17	Cysteine and methionine metabolism	map00270	7	ec:2.1.1.5 - S-methyltransferase, ec:1.1.1.37 - dehydrogenase, ec:4.2.1.109 - 1-phosphate dehydratase, ec:2.1.1.37 - (cytosine-5-)-methyltransferase, ec:1.14.17.4 - oxidase, ec:3.1.3.77 - synthase, ec:5.3.2.5 - enolase	9	LOC4332095, LOC4327423, LOC4350524, LOC4332128, LOC4334435, LOC4330873, LOC4350524, LOC107277360, LOC4350524
18	Th1 and Th2 cell differentiation	map04658	1	ec:3.1.3.16 - phosphatase	9	LOC107275989, LOC4326496, LOC4335297, LOC4334153, LOC4324753, LOC4351304, LOC4329882, LOC4330100, LOC4348468
19	T cell receptor signaling pathway	map04660	1	ec:3.1.3.16 - phosphatase	9	LOC107275989, LOC4326496, LOC4335297, LOC4334153, LOC4324753, LOC4351304, LOC4329882, LOC4330100, LOC4348468
20	Porphyrin and chlorophyll metabolism	map00860	7	ec:1.14.13.122 - oxygenase, ec:3.1.1.14 - CLH, ec:1.2.1.70 - reductase, ec:1.3.3.3 - oxidase, ec:3.2.1.31 - beta-glucuronide glucuronohydrolase glucuronidase, ec:1.1.1.294 - b reductase, ec:6.1.1.17 - ligase	8	LOC4334498, LOC4348648, LOC4349044, LOC4351951, LOC4332033, LOC107280215, LOC4325167, LOC4348464
21	Diterpenoid biosynthesis	map0	6	ec:5.5.1.13 - diphosphate synthase, ec:4.2.3.30 - synthase, ec:4.2.3.29 - synthase, ec:1.14.11.13 - 2beta-dioxygenase, ec:4.2.3.33 - synthase, ec:4.2.3.34 - synthase	8	LOC9266189, LOC4329729, LOC4329728, LOC4352248, LOC4339600, LOC9266251, LOC4350502, LOC4350502

		0 9 0 4				
22	Riboflavin metabolism	map00740	2	ec:2.7.7.2 - synthetase, ec:3.1.3.2 - phosphatase	8	LOC4334460, LOC4332737, LOC9269151, LOC4338023, LOC4334778, LOC4338021, LOC9270081, LOC4327297
23	Glycine, serine and threonine metabolism	map00260	6	ec:2.1.1.5 - S-methyltransferase, ec:1.14.15.7 - monooxygenase, ec:4.2.1.20 - synthase, ec:4.3.1.19 - ammonia-lyase, ec:1.4.3.21 - oxidase, ec:3.1.3.3 - phosphatase	7	LOC4332095, LOC9272377, LOC4334425, LOC4333898, LOC4335335, LOC107276105, LOC4330100
24	Glycerolipid metabolism	map00561	4	ec:2.3.1.20 - O-acyltransferase, ec:3.1.1.23 - lipase, ec:2.7.7.9 - uridylyltransferase, ec:2.3.1.158 - acyltransferase	7	LOC4325602, LOC4338045, LOC9270328, LOC107275275, LOC4328091, LOC4326201, LOC4347190
25	Streptomycin biosynthesis	map00521	5	ec:5.1.3.13 - 3,5-epimerase, ec:2.7.1.1 - hexokinase type IV glucokinase, ec:2.7.1.2 - glucokinase (phosphorylating), ec:5.5.1.4 - synthase, ec:1.1.1.133 - reductase	7	LOC9267465, LOC107275630, LOC4324666, LOC107275630, LOC4324666, LOC4331917, LOC9267465

26	alpha-Linolenic acid metabolism	map00592	4	ec:4.2.1.92 - dehydratase, ec:1.13.11.12 - 13S-lipoxygenase, ec:1.3.3.6 - oxidase, ec:3.1.1.32 - A1	6	LOC4332121, LOC4352505, LOC4352509, LOC4340986, LOC107276812, LOC4327832
27	Glycerophospholipid metabolism	map00564	4	ec:3.1.4.4 - D, ec:3.6.1.16 - diphosphatase, ec:3.1.4.3 - C, ec:3.1.1.32 - A1	6	LOC4334732, LOC4341468, LOC4346295, LOC4329953, LOC107276812, LOC4327832
28	Pyruvate metabolism	map00620	5	ec:4.1.1.32 - carboxykinase (GTP), ec:2.3.1.12 - acetyltransferase, ec:1.1.1.37 - dehydrogenase, ec:4.4.1.5 - lyase, ec:4.1.1.31 - carboxylase	5	LOC4346699, LOC4343003, LOC4327423, LOC107276896, LOC4346699
29	Caffeine metabolism	map00232	1	ec:2.3.1.5 - N-acetyltransferase	5	LOC4334126, LOC4347190, LOC107276987, LOC4341999, LOC4344443
30	Nitrotoluene degradation	map0	1	ec:2.3.1.5 - N-acetyltransferase	5	LOC4334126, LOC4347190, LOC107276987, LOC4341999, LOC4344443

		00633				
31	Alanine, aspartate and glutamate metabolism	map00250	5	ec:6.3.5.5 - synthase (glutamine-hydrolysing), ec:1.4.1.14 - synthase (NADH), ec:4.3.2.1 - lyase, ec:6.3.4.4 - synthase, ec:3.5.1.1 - asparaginase II	5	LOC4330474, LOC4324398, LOC107275838, LOC4331792, LOC107280261
32	Lysine degradation	map00310	1	ec:2.1.1.43 - N-methyltransferase	5	LOC4330010, LOC4329647, LOC4344819, LOC4326952, LOC4346404
33	Sulfur metabolism	map00920	3	ec:3.1.3.7 - nucleotidase, ec:2.7.7.4 - adenylyltransferase, ec:2.7.1.25 - kinase	5	LOC4351686, LOC4351685, LOC4334932, LOC4334932, LOC9272308
34	Methane metabolism	map00680	4	ec:1.1.1.37 - dehydrogenase, ec:3.1.3.3 - phosphatase, ec:4.1.1.31 - carboxylase, ec:2.7.1.11 - phosphohexokinase	5	LOC4327423, LOC4330100, LOC4346699, LOC4327196, LOC4338034

35	Citrate cycle (TCA cycle)	map00020	5	ec:1.3.5.1 - dehydrogenase, ec:4.1.1.32 - carboxykinase (GTP), ec:2.3.1.12 - acetyltransferase, ec:2.3.3.8 - citrate synthase, ec:1.1.1.37 - dehydrogenase	5	LOC4343404, LOC4346699, LOC4343003, LOC9272295, LOC4327423
36	Neomycin, kanamycin and gentamicin biosynthesis	map00524	2	ec:2.7.1.1 - hexokinase type IV glucokinase, ec:2.7.1.2 - glucokinase (phosphorylating)	4	LOC107275630, LOC4324666, LOC107275630, LOC4324666
37	beta-Alanine metabolism	map00410	3	ec:3.1.2.4 - hydrolase, ec:1.4.3.21 - oxidase, ec:1.5.3.14 - oxidase (propane-1,3-diamine-forming)	4	LOC4351929, LOC4335335, LOC107276105, LOC4331922
38	Phenylalanine, tyrosine and tryptophan biosynthesis	map00400	4	ec:1.1.1.25 - dehydrogenase, ec:4.2.1.10 - dehydratase, ec:1.1.1.282 - dehydrogenase, ec:4.2.1.20 - synthase	4	LOC4325444, LOC4325444, LOC4325444, LOC4334425
39	Drug metabolism -	map0	2	ec:1.14.13.8 - monooxygenase, ec:2.5.1.18 - transferase	4	LOC4325178, LOC107275877, LOC4348461, LOC4348549

	cytochrome P450	09 82				
40	Zeatin biosynthesis	map00 908	2	ec:2.5.1.75 - dimethylallyltransferase, ec:1.5.99.12 - dehydrogenase	4	LOC4334529, LOC4339535, LOC107275724, LOC4327333
41	Carbon fixation pathways in prokaryotes	map00 720	4	ec:1.3.5.1 - dehydrogenase, ec:2.3.3.8 - citrate synthase, ec:1.1.1.37 - dehydrogenase, ec:4.1.1.31 - carboxylase	4	LOC4343404, LOC9272295, LOC4327423, LOC4346699
42	Ascorbate and aldarate metabolism	map00 053	3	ec:2.7.7.64 - uridylyltransferase, ec:1.6.5.4 - reductase (NADH), ec:1.1.1.22 - 6-dehydrogenase	4	LOC4328091, LOC4326201, LOC4346299, LOC4333410
43	Carbon fixation in photosynthetic organisms	map00 710	4	ec:2.7.2.3 - kinase, ec:2.7.1.19 - phosphopentokinase, ec:1.1.1.37 - dehydrogenase, ec:4.1.1.31 - carboxylase	4	LOC4348728, LOC4330413, LOC4327423, LOC4346699

44	Glutathione metabolism	map00480	2	ec:2.5.1.18 - transferase, ec:1.8.1.7 - reductase	3	LOC4348461, LOC4348549, LOC4329999
45	Cyanoamino acid metabolism	map00460	2	ec:3.2.1.21 - gentiobiase, ec:3.5.1.1 - asparaginase II	3	LOC9270758, LOC4338560, LOC107280261
46	Selenocompound metabolism	map00450	2	ec:2.7.7.4 - adenylyltransferase, ec:6.1.1.10 - ligase	3	LOC4334932, LOC4332018, LOC4348562
47	Steroid degradation	map00984	1	ec:1.1.1.145 - dehydrogenase	3	LOC107278880, LOC4333070, LOC4346551
48	Valine, leucine and isoleucine	map0	3	ec:4.2.1.35 - dehydratase, ec:4.3.1.19 - ammonia-lyase, ec:4.2.1.33 - dehydratase	3	LOC4330192, LOC4333898, LOC4330192

	biosynthesis	0290				
49	Sphingolipid metabolism	map00600	2	ec:2.3.1.24 - N-acyltransferase, ec:4.1.2.27 - aldolase	3	LOC4332339, LOC4329785, LOC4326459
50	Tyrosine metabolism	map00350	2	ec:1.10.3.1 - oxidase, ec:1.4.3.21 - oxidase	3	LOC9271853, LOC4335335, LOC107276105
51	Arginine and proline metabolism	map00330	2	ec:1.5.3.14 - oxidase (propane-1,3-diamine-forming), ec:1.14.11.2 - 4-dioxygenase	3	LOC4331922, LOC4331736, LOC4334196
52	Isoquinoline alkaloid biosynthesis	map00950	2	ec:1.10.3.1 - oxidase, ec:1.4.3.21 - oxidase	3	LOC9271853, LOC4335335, LOC107276105

53	Steroid hormone biosynthesis	map00140	1	ec:1.1.1.145 - dehydrogenase	3	LOC107278880, LOC4333070, LOC4346551
54	Ether lipid metabolism	map00565	2	ec:3.1.4.4 - D, ec:3.1.4.3 - C	3	LOC4334732, LOC4341468, LOC4329953
55	Terpenoid backbone biosynthesis	map00900	3	ec:2.7.4.2 - kinase, ec:4.6.1.12 - 2,4-cyclodiphosphate synthase, ec:2.5.1.29 - diphosphate synthase	3	LOC4332275, LOC4330320, LOC4323939
56	Cutin, suberine and wax biosynthesis	map00073	2	ec:2.3.1.20 - O-acyltransferase, ec:2.3.1.75 - O-fatty-acyltransferase	3	LOC4325602, LOC4338045, LOC4325602
57	Pentose phosphate pathway	map0	2	ec:5.3.1.9 - isomerase, ec:2.7.1.11 - phosphohexokinase	3	LOC4340677, LOC4327196, LOC4338034

		0 0 3 0				
58	Glyoxylate and dicarboxylate metabolism	map00630	2	ec:3.5.1.9 - kynurenine formamidase, ec:1.1.1.37 - dehydrogenase	2	LOC4341616, LOC4327423
59	Tryptophan metabolism	map00380	2	ec:3.5.1.9 - kynurenine formamidase, ec:2.6.1.27 - transaminase	2	LOC4341616, LOC4325198
60	Biosynthesis of unsaturated fatty acids	map01040	2	ec:1.3.3.6 - oxidase, ec:4.2.1.134 - (3R)-3-hydroxyacyl-CoA dehydratase	2	LOC4340986, LOC4324521
61	Metabolism of xenobiotics by cytochrome P450	map00980	1	ec:2.5.1.18 - transferase	2	LOC4348461, LOC4348549

62	Phenylalanine metabolism	map00360	1	ec:1.4.3.21 - oxidase	2	LOC4335335, LOC107276105
63	mTOR signaling pathway	map04150	1	ec:2.7.11.24 - protein kinase	2	LOC4344698, LOC4342017
64	Tropane, piperidine and pyridine alkaloid biosynthesis	map00960	1	ec:1.4.3.21 - oxidase	2	LOC4335335, LOC107276105
65	Oxidative phosphorylation	map00190	2	ec:3.6.1.1 - diphosphatase, ec:1.3.5.1 - dehydrogenase	2	LOC4326846, LOC4343404
66	Polyketide sugar unit	map0	2	ec:5.1.3.13 - 3,5-epimerase, ec:1.1.1.133 - reductase	2	LOC9267465, LOC9267465

	biosynthesis	05 23				
67	Fatty acid degradation	map00071	2	ec:1.14.15.3 - 1-monooxygenase, ec:1.3.3.6 - oxidase	2	LOC4331756, LOC4340986
68	Linoleic acid metabolism	map00591	1	ec:1.13.11.12 - 13S-lipoxygenase	2	LOC4352505, LOC4352509
69	Fatty acid biosynthesis	map00061	2	ec:2.3.1.85 - synthase, ec:1.1.1.100 - reductase	2	LOC4335609, LOC4335609
70	Nicotinate and nicotinamide metabolism	map00760	2	ec:2.7.7.1 - adenylyltransferase, ec:2.7.1.23 - kinase	2	LOC4325639, LOC4327845

71	Arginine biosynthesis	map00220	2	ec:4.3.2.1 - lyase, ec:3.5.1.16 - deacetylase	2	LOC107275838, LOC4330380
72	Arachidonic acid metabolism	map00590	2	ec:1.14.15.3 - 1-monooxygenase, ec:5.3.99.3 - synthase	2	LOC4331756, LOC4335293
73	One carbon pool by folate	map00670	2	ec:1.5.1.3 - reductase, ec:2.1.1.45 - synthase	2	LOC4350527, LOC4350527
74	Nitrogen metabolism	map00910	2	ec:1.4.1.14 - synthase (NADH), ec:4.2.1.1 - anhydrase	2	LOC4324398, LOC4347247
75	Ubiquinone and other terpenoid-	map0	2	ec:2.2.1.9 - synthase, ec:4.2.99.20 - synthase	2	LOC4329786, LOC4329786

	quinone biosynthesis	01300				
76	Monobactam biosynthesis	map00261	1	ec:2.7.7.4 - adenylyltransferase	1	LOC4334932
77	Stilbenoid, diarylheptanoid and gingerol biosynthesis	map00945	1	ec:2.3.1.211 - synthase	1	LOC4342896
78	Carotenoid biosynthesis	map00906	1	ec:1.13.11.69 - synthase	1	LOC4326177
79	Fatty acid elongation	map00062	1	ec:4.2.1.134 - (3R)-3-hydroxyacyl-CoA dehydratase	1	LOC4324521

80	Valine, leucine and isoleucine degradation	map00280	1	ec:3.1.2.4 - hydrolase	1	LOC4351929
81	N-Glycan biosynthesis	map00510	1	ec:3.2.1.84 - 1,3-alpha-glucosidase	1	LOC4332068
82	Histidine metabolism	map00340	1	ec:5.3.1.16 - isomerase	1	LOC9270806
83	Folate biosynthesis	map00790	1	ec:1.5.1.3 - reductase	1	LOC4350527
84	Flavone and flavonol	map0	1	ec:3.2.1.31 - beta-glucuronide glucuronohydrolase glucuronidase	1	LOC4332033

	biosynthesis	09444				
85	Glycosphingolipid biosynthesis - globo and isoglobo series	map00603	1	ec:2.4.1.69 - 1 galactoside alpha-(1,2)-fucosyltransferase	1	LOC4330826
86	Indole alkaloid biosynthesis	map00901	1	ec:4.3.3.2 - synthase	1	LOC4345695
87	Caprolactam degradation	map00930	1	ec:1.14.15.3 - 1-monooxygenase	1	LOC4331756
88	Biotin metabolism	map00780	1	ec:1.1.1.100 - reductase	1	LOC4335609

89	Lysine biosynthesis	map00300	1	ec:4.1.1.20 - decarboxylase	1	LOC4329234
90	Pantothenate and CoA biosynthesis	map00770	1	ec:2.7.8.7 - synthase	1	LOC4351429
91	C5-Branched dibasic acid metabolism	map00660	1	ec:4.2.1.35 - dehydratase	1	LOC4330192
92	Butanoate metabolism	map00650	1	ec:1.3.5.1 - dehydrogenase	1	LOC4343404
93	Steroid biosynthesis	map0	1	ec:5.5.1.9 - cycloisomerase	1	LOC9270960

		0 1 0 0				
9 4	Glycosam inoglycan degradati on	m a p 0 0 5 3 1	1	ec:3.2.1.31 - beta-glucuronide glucuronohydrolase glucuronidase	1	LOC4332033
9 5	Other glycan degradati on	m a p 0 0 5 1 1	1	ec:3.2.1.96 - endo-beta-N-acetylglucosaminidase	1	LOC4338487
9 6	Propanoat e metabolis m	m a p 0 0 6 4 0	1	ec:3.1.2.4 - hydrolase	1	LOC4351929
9 7	Glycosphi ngolipid biosynthe sis - lacto and neolacto series	m a p 0 0 6 0 1	1	ec:2.4.1.69 - 1 galactoside alpha-(1,2)-fucosyltransferase	1	LOC4330826

**32<br>4**	**170 unique enzymes**	**74<br>4**	**311 unique genes**

Table S6. Pathways, enzymes and sequences (genes) recorded by functional-annotation analysis of 1479 TAVs for TGW trait.

No	Pathway	Pathway ID	#Enzs in Pathway	Enzyme	#Seqs of Enzyme	Seqs
1	Purine metabolism	map00230	3	ec:3.6.1.15 - phosphatase, ec:3.6.1.3 - adenylpyrophosphatase, ec:2.7.7.6 - RNA polymerase	54	LOC4335000, LOC107275844, LOC4336770, LOC4345008, LOC4341902, LOC4352060, LOC4343066, LOC4327122, LOC4326284, LOC4329409, LOC4337825, LOC9266862, LOC4337589, LOC4342943, LOC4343339, LOC4330083, LOC4329095, LOC4348944, LOC4326314, LOC4326797, LOC4326410, LOC4329117, LOC4352796, LOC4352456, LOC4332469, LOC107280174, LOC4328702, LOC107279988, LOC4335338, LOC107279989, LOC9268713, LOC4330342, LOC4339511, LOC4350530, LOC4342943, LOC4343339, LOC107275844, LOC4336770, LOC4341902, LOC4352060, LOC4329095, LOC4327122, LOC4326410, LOC4352796, LOC4352456, LOC4332469, LOC4328702, LOC4326284, LOC4329409, LOC4337825, LOC4330342, LOC4339511, LOC4350530, LOC4337831
2	Thiamine metabolism	map00730	3	ec:3.6.1.15 - phosphatase, ec:3.1.3.2 - phosphatase, ec:2.7.6.2 - diphosphokinase	38	LOC4335000, LOC107275844, LOC4336770, LOC4345008, LOC4341902, LOC4352060, LOC4343066, LOC4327122, LOC4326284, LOC4329409, LOC4337825, LOC9266862, LOC4337589, LOC4342943, LOC4343339, LOC4330083, LOC4329095, LOC4348944, LOC4326314, LOC4326797, LOC4326410, LOC4329117, LOC4352796, LOC4352456, LOC4332469, LOC107280174, LOC4328702, LOC107279988, LOC4335338, LOC107279989, LOC9268713, LOC4330342, LOC4339511, LOC4350530, LOC4344302, LOC4352613, LOC4333280, LOC4326817
3	Biosynthesis of antibiotics	map01130	12	ec:4.1.1.32 - carboxykinase (GTP), ec:3.1.3.3 - phosphatase, ec:5.1.3.13 - 3,5-epimerase, ec:1.1.1.25 - dehydrogenase, ec:2.7.7.64 - uridylyltransferase, ec:4.2.1.10 - dehydratase, ec:2.4.2.18 - phosphoribosyltransferase, ec:5.4.99.5 - mutase, ec:1.1.1.282 - dehydrogenase, ec:2.7.7.9 - uridylyltransferase, ec:1.1.1.133 - reductase,	15	LOC4346699, LOC4330100, LOC9267465, LOC4325444, LOC4325446, LOC4328091, LOC4325444, LOC4325446, LOC4331468, LOC4352624, LOC4325444, LOC4325446, LOC4328091, LOC9267465, LOC4341770

				ec:3.1.3.11 - hexose diphosphatase		
4	Aminobenzoate degradation	map00627	1	ec:3.1.3.41 - nitrophenyl phosphatase	15	LOC4335297, LOC4333280, LOC4325448, LOC4332509, LOC4344302, LOC4341770, LOC4329347, LOC4326496, LOC107275373, LOC4349283, LOC4352613, LOC4337619, LOC107276788, LOC4330100, LOC4351686
5	Starch and sucrose metabolism	map00500	9	ec:3.1.3.24 - phosphatase, ec:3.2.1.21 - gentiobiase, ec:3.2.1.4 - endo-1,4-beta-D-glucanase, ec:3.2.1.26 - invertase, ec:3.2.1.39 - endo-1,3-beta-D-glucosidase, ec:2.4.1.34 - synthase, ec:2.7.7.9 - uridylyltransferase, ec:2.4.1.12 - synthase (UDP-forming), ec:2.4.1.1 - phosphorylase	11	LOC4325448, LOC4325830, LOC4347440, LOC4347441, LOC4337698, LOC4335790, LOC4325830, LOC9271759, LOC4328091, LOC107276048, LOC4352773
6	Phenylpropanoid biosynthesis	map00940	2	ec:3.2.1.21 - gentiobiase, ec:1.11.1.7 - lactoperoxidase	9	LOC4325830, LOC4347440, LOC4347441, LOC4328841, LOC4337892, LOC4328842, LOC4326027, LOC9272450, LOC4344281
7	Amino sugar and nucleotide sugar metabolism	map00520	7	ec:5.1.3.2 - 4-epimerase, ec:1.1.1.22 - 6-dehydrogenase, ec:3.2.1.55 - end alpha-L-arabinofuranosidase, ec:2.7.7.64 - uridylyltransferase, ec:2.4.1.43 - 4-alpha-galacturonosyltransferase, ec:3.2.1.14 - ChiC, ec:2.7.7.9 - uridylyltransferase	9	LOC9266692, LOC4333410, LOC4341521, LOC107276997, LOC4328091, LOC4352627, LOC4327951, LOC4349267, LOC4328091
8	Drug metabolism - other enzymes	map00983	2	ec:2.5.1.18 - transferase, ec:3.1.1.1 - ali-esterase	9	LOC4325437, LOC4337622, LOC4351775, LOC4332758, LOC9270328, LOC4333799, LOC4339700, LOC4346920, LOC4330908
9	Phenylalanine, tyrosine and	map00400	5	ec:1.1.1.25 - dehydrogenase, ec:4.2.1.10 - dehydratase, ec:2.4.2.18 -	8	LOC4325444, LOC4325446, LOC4325444, LOC4325446, LOC4331468, LOC4352624, LOC4325444, LOC4325446

	tryptophan biosynthesis			phosphoribosyltransferase, ec:5.4.99.5 - mutase, ec:1.1.1.282 - dehydrogenase		
10	Th1 and Th2 cell differentiation	map04658	1	ec:3.1.3.16 - phosphatase	8	LOC4329347, LOC4326496, LOC4335297, LOC107275373, LOC4349283, LOC4337619, LOC4332509, LOC4330100
11	T cell receptor signaling pathway	map04660	1	ec:3.1.3.16 - phosphatase	8	LOC4329347, LOC4326496, LOC4335297, LOC107275373, LOC4349283, LOC4337619, LOC4332509, LOC4330100
12	Aminoacyl-tRNA biosynthesis	map00970	6	ec:6.1.1.18 - ligase, ec:6.1.1.4 - ligase, ec:6.1.1.5 - ligase, ec:6.1.1.9 - ligase, ec:6.1.1.21 - ligase, ec:6.1.1.14 - ligase	7	LOC4325653, LOC4346920, LOC4330908, LOC4332758, LOC4333799, LOC4337830, LOC4339847
13	Galactose metabolism	map00052	5	ec:5.1.3.2 - 4-epimerase, ec:3.2.1.23 - lactase (ambiguous), ec:3.2.1.26 - invertase, ec:2.7.7.64 - uridylyltransferase, ec:2.7.7.9 - uridylyltransferase	6	LOC9266692, LOC4327808, LOC4325748, LOC4335790, LOC4328091, LOC4328091
14	Riboflavin metabolism	map00740	4	ec:1.5.1.38 - reductase (NADPH), ec:3.5.4.26 - deaminase, ec:1.5.1.39 - reductase [NAD(P)H], ec:3.1.3.2 - phosphatase	6	LOC4325401, LOC9272088, LOC4325401, LOC4344302, LOC4352613, LOC4333280
15	Glutathione metabolism	map00480	3	ec:2.5.1.18 - transferase, ec:1.8.5.1 - dehydrogenase (ascorbate), ec:6.3.2.2 - ligase	5	LOC4325437, LOC4337622, LOC4351775, LOC4337622, LOC4337696
16	Drug metabolism - cytochrome P450	map00982	2	ec:1.14.13.8 - monooxygenase, ec:2.5.1.18 - transferase	4	LOC9267722, LOC4325437, LOC4337622, LOC4351775
17	Diterpenoid biosynthesis	map00904	4	ec:4.2.3.35 - synthase, ec:5.5.1.13 - diphosphate	4	LOC4335094, LOC9266189, LOC4339600, LOC4335090

				synthase, ec:1.14.11.13 - 2beta-dioxygenase, ec:5.5.1.14 - synthase		
18	Methane metabolism	map00680	4	ec:3.1.3.3 - phosphatase, ec:4.1.1.31 - carboxylase, ec:4.1.1.25 - decarboxylase, ec:3.1.3.11 - hexose diphosphatase	4	LOC4330100, LOC4346699, LOC4325604, LOC4341770
19	Pentose and glucuronate interconversions	map00040	4	ec:3.2.1.15 - pectin depolymerase, ec:1.1.1.22 - 6-dehydrogenase, ec:2.7.7.64 - uridylyltransferase, ec:2.7.7.9 - uridylyltransferase	4	LOC4339708, LOC4333410, LOC4328091, LOC4328091
20	Glycerophospholipid metabolism	map00564	4	ec:1.1.1.8 - dehydrogenase (NAD+), ec:1.1.5.3 - dehydrogenase, ec:3.1.4.3 - C, ec:1.1.1.94 - dehydrogenase [NAD(P)+]	4	LOC4335210, LOC4335210, LOC4337686, LOC4335210
21	Inositol phosphate metabolism	map00562	4	ec:3.1.4.11 - phospholipase C, ec:3.1.4.3 - C, ec:3.1.3.36 - 5-phosphatase, ec:2.7.1.68 - 5-kinase	4	LOC4337686, LOC4337686, LOC107276788, LOC9267438
22	Cyanoamino acid metabolism	map00460	1	ec:3.2.1.21 - gentiobiase	3	LOC4325830, LOC4347440, LOC4347441
23	Glycerolipid metabolism	map00561	3	ec:2.3.1.20 - O-acyltransferase, ec:3.1.1.23 - lipase, ec:2.7.7.9 - uridylyltransferase	3	LOC4325603, LOC9270328, LOC4328091
24	Metabolism of xenobiotics by cytochrome P450	map00980	1	ec:2.5.1.18 - transferase	3	LOC4325437, LOC4337622, LOC4351775

25	Phosphatidy linositol signaling system	map04070	3	ec:3.1.4.11 - phospholipase C, ec:3.1.3.36 - 5-phosphatase, ec:2.7.1.68 - 5-kinase	3	LOC4337686, LOC107276788, LOC9267438
26	Ascorbate and aldarate metabolism	map00053	3	ec:1.1.1.22 - 6-dehydrogenase, ec:1.8.5.1 - dehydrogenase (ascorbate), ec:2.7.7.64 - uridylyltransferase	3	LOC4333410, LOC4337622, LOC4328091
27	Other glycan degradation	map00511	2	ec:3.2.1.51 - alpha-fucosidase, ec:3.2.1.23 - lactase (ambiguous)	3	LOC4336650, LOC4327808, LOC4325748
28	Glycolysis / Gluconeogen esis	map00010	2	ec:4.1.1.32 - carboxykinase (GTP), ec:3.1.3.11 - hexose diphosphatase	2	LOC4346699, LOC4341770
29	Pyruvate metabolism	map00620	2	ec:4.1.1.32 - carboxykinase (GTP), ec:4.1.1.31 - carboxylase	2	LOC4346699, LOC4346699
30	Sulfur metabolism	map00920	2	ec:3.1.3.7 - nucleotidase, ec:2.8.1.1 - sulfurtransferase	2	LOC4351686, LOC4352752
31	Carotenoid biosynthesis	map00906	2	ec:1.13.11.69 - synthase, ec:1.13.11.51 - dioxygenase	2	LOC4327933, LOC107275432
32	Glycosphing olipid biosynthesis - ganglio series	map00604	1	ec:3.2.1.23 - lactase (ambiguous)	2	LOC4327808, LOC4325748
33	Sphingolipid metabolism	map00600	1	ec:3.2.1.23 - lactase (ambiguous)	2	LOC4327808, LOC4325748
34	Tyrosine metabolism	map00350	2	ec:5.2.1.2 - isomerase, ec:4.1.1.25 - decarboxylase	2	LOC4351775, LOC4325604
35	Cutin, suberine and wax biosynthesis	map00073	2	ec:2.3.1.20 - O-acyltransferase, ec:2.3.1.75 - O-fatty-acyltransferase	2	LOC4325603, LOC4325603

36	Polyketide sugar unit biosynthesis	map00523	2	ec:5.1.3.13 - 3,5-epimerase, ec:1.1.1.133 - reductase	2	LOC9267465, LOC9267465
37	Glycosamino glycan degradation	map00531	1	ec:3.2.1.23 - lactase (ambiguous)	2	LOC4327808, LOC4325748
38	Carbon fixation in photosynthet ic organisms	map00710	2	ec:4.1.1.31 - carboxylase, ec:3.1.3.11 - hexose diphosphatase	2	LOC4346699, LOC4341770
39	Streptomyci n biosynthesis	map00521	2	ec:5.1.3.13 - 3,5-epimerase, ec:1.1.1.133 - reductase	2	LOC9267465, LOC9267465
40	Glyoxylate and dicarboxylat e metabolism	map00630	1	ec:1.2.3.5 - oxidase	1	LOC4325521
41	Biotin metabolism	map00780	1	ec:2.6.1.62 - transaminase	1	LOC4336983
42	Pantothenate and CoA biosynthesis	map00770	1	ec:2.1.2.11 - hydroxymethyltransferase	1	LOC4326739
43	Selenocomp ound metabolism	map00450	1	ec:2.1.1.12 - S-methyltransferase	1	LOC4337554
44	alpha-Linolenic acid metabolism	map00592	1	ec:4.2.1.92 - dehydratase	1	LOC4328742
45	Steroid degradation	map00984	1	ec:1.3.99.5 - 4-dehydrogenase (acceptor)	1	LOC4336740
46	Fatty acid elongation	map00062	1	ec:4.2.1.134 - (3R)-3-hydroxyacyl-CoA dehydratase	1	LOC4324520

47	Styrene degradation	map00643	1	ec:5.2.1.2 - isomerase	1	LOC4351775
48	Ether lipid metabolism	map00565	1	ec:3.1.4.3 - C	1	LOC4337686
49	Biosynthesis of unsaturated fatty acids	map01040	1	ec:4.2.1.134 - (3R)-3-hydroxyacyl-CoA dehydratase	1	LOC4324520
50	Zeatin biosynthesis	map00908	1	ec:2.5.1.75 - dimethylallyltransferase	1	LOC107275425
51	Ubiquinone and other terpenoid-quinone biosynthesis	map00130	1	ec:1.6.5.2 - dehydrogenase (quinone)	1	LOC4325401
52	Butanoate metabolism	map00650	1	ec:2.6.1.96 - transaminase	1	LOC4336983
53	Oxidative phosphorylation	map00190	1	ec:3.6.1.1 - diphosphatase	1	LOC4329579
54	Cysteine and methionine metabolism	map00270	1	ec:6.3.2.2 - ligase	1	LOC4337696
55	Carbon fixation pathways in prokaryotes	map00720	1	ec:4.1.1.31 - carboxylase	1	LOC4346699
56	Glycine, serine and threonine metabolism	map00260	1	ec:3.1.3.3 - phosphatase	1	LOC4330100
57	Alanine, aspartate and	map00250	1	ec:2.6.1.96 - transaminase	1	LOC4336983

	glutamate metabolism					
58	Pentose phosphate pathway	map00030	1	ec:3.1.3.11 - hexose diphosphatase	1	LOC4341770
59	Pyrimidine metabolism	map00240	1	ec:2.7.7.6 - RNA polymerase	1	LOC4337831
60	Fructose and mannose metabolism	map00051	1	ec:3.1.3.11 - hexose diphosphatase	1	LOC4341770
61	Isoquinoline alkaloid biosynthesis	map00950	1	ec:4.1.1.25 - decarboxylase	1	LOC4325604
62	Citrate cycle (TCA cycle)	map00020	1	ec:4.1.1.32 - carboxykinase (GTP)	1	LOC4346699
			**143**	**82 unique enzymes**	**297**	**120 unique enzymes**

Table S7. Candidate genes for the three agronomic traits, heading date, tiller number and 1000-grain weight.

Heading Date (Hd) Trait								
**Gene***	**Gene symbol**	**Also known as**	**Description**	**Locus tag**	**Chr #**	**Exon count**	**Length (nt)**	**Protein length (aa)**
DTH2	LOC4330574	-	zinc finger protein CONSTANS-LIKE 9	Os02g0724000	2	7	4953	407
Hd16	LOC4334396	EL1	casein kinase 1-like protein HD16	Os03g0793500	3	18	7380	707
Hd1	LOC4340746	SE1	zinc finger protein HD1-like	Os06g0275000	6	2	2297	395
Hd3a	LOC4340185	-	protein HEADING DATE 3A-like	Os06g0157700	6	4	2407	179
Hd3b	LOC4340184	RFT1	protein RICE FLOWERING LOCUS T 1-like	Os06g0157500	6	4	1792	178
Hd5	LOC4344784	DTH8	nuclear transcription factor Y subunit B-11-like	Os08g0174500	8	1	1704	297
Ehd3	LOC4344443	-	PHD finger protein EHD3-like	Os08g0105000	8	6	3730	563
Ehd1	LOC107276289	ef1	two-component response regulator ORR30	Os10g0463400	10	5	5616	341
Ehd2	LOC4348644	RID1	protein indeterminate-domain 12	Os10g0419200	10	3	3771	475

Tiller Number at Maturity (T) Trait								
**Gene**	**Gene symbol**	**Also known as**	**Description**	**Protein name**	**Chr #**	**Exon count**	**Length (nt)**	**Protein length (aa)**
D10	LOC4326177	-	carotenoid cleavage dioxygenase 8 homolog B, chloroplastic	Os01g0746400	1	5	3825	569
RCN1	LOC4332449	WBC5	ABC transporter G family member 5-like	Os03g0281900	3	1	2910	787
D14	LOC4331983	HTD2-D88	strigolactone esterase D14-like	Os03g0203200	3	2	1897	318
OsTB1	LOC4333856	-	transcription factor TB1-like	Os03g0706500	3	1	2151	388
D17	LOC4336591	HTD1	carotenoid cleavage dioxygenase 7, chloroplastic-like	Os04g0550600	4	7	2885	609
DWARF3	LOC4339885	-	ent-kaurenoic acid oxidase-like	Os06g0110000	6	7	5785	506
D3	LOC9272469	-	F-box/LRR-repeat MAX2 homolog	Os06g0154200	6	2	3970	720
MOC1	LOC107278653	-	protein MONOCULM 1	Os06g0610350	6	1	2817	441
OsSPL14	LOC4345998	-	squamosa promoter-binding-like protein 14	Os08g0509600	8	3	4393	418
D53	LOC4349543	DWARF53	Protein DWARF 53	Os11g0104300	11	3	5817	1131

1000-Grain Weight (TGW) Trait								
**Gene**	**Gene symbol**	**Also known as**	**Description**	**Protein name**	**Chr #**	**Exon count**	**Length (nt)**	**Protein length (aa)**
Gn1a	LOC4327333	CKX2	cytokinin dehydrogenase 2-like	Os01g0197700	1	4	5380	565
GW2	LOC4328856	-	E3 ubiquitin-protein ligase GW2	Os02g0244100	2	8	6907	425
GS3	LOC9269602	-	keratin-associated protein 5-5	Os03g0407400	3	5	6009	201
GIF1	LOC4335790	WB1; CIN2	beta-fructofuranosidase, insoluble isoenzyme 2-like	Os04g0413500	4	7	4876	598

GW5	LOC4338011	-	protein IQ-DOMAIN 14	Os05g0187500	5	3	2162	470

***DTH:** Days to Heading, **Hd:** Heading date, **Ehd:** Early heading date, **D:** Dwarf, **RCN:** Reduce Culm Number, **HTD:** HIGH-TILLERING DWARF, **OsTB:** TEOSINTE BRANCHED, **MOC:** MonoCulm**, Gn:** Grain productivity, **GW:** Grain width, **GS:** Grain size/shape, **GIF:** Grain incomplete filling, **CKX:** Cytokinin dehydrogenase, **EL:** Early flowering, **SE:** Photoperiod Sensitivity, **WBC:** White-brown complex homolog protein; **Chr #:** Chromosome's number, **nt:** Nucleotide**, aa:** Amino Acid.

Table S8. Summary of the value of each agronomic trait and its bulks.

Traits	Trait value in population	Unit	Bulks	ID	Trait value in bulks	Gap between bulks values
**Heading date (Hd)**	59-98	Days	**Low value (L)**	**HdL**	59-62	26 Days
			**High value (H)**	**HdH**	88-98	
**Tiller number at maturity (T)**	3-18	Tiller count	**Low value (L)**	**TL**	3-4	8 Tiller
			**High value (H)**	**TH**	12-18	
**1000-Grain Weight (TGW)**	21-43	Gram	**Low value (L)**	**TGWL**	21-25	11 Gram
			**High value (H)**	**TGWH**	36-43	

Table S9. Phenotypic data of BC2F9 and BC2F10 populations of total of 285 Backcross Inbred Lines (BILs).

No	Name	BC2F9 (2012)			BC2F10 (2013)			No	Name	BC2F9 (2012)			BC2F10 (2013)		
		Hd (days)	T (tiller number)	TGW (gram)	Hd (days)	T (tiller number)	TGW (gram)			Hd (days)	T (tiller number)	TGW (gram)	Hd (days)	T (tiller number)	TGW (gram)
1	97-2-4	60	6	29.2	69	6	27.26	144	121-5-2	73	11	28.0	71	7	27.1
2	121-1-10	58	8	29.0	60	5	31.8	145	101-5	73	6	32.2	82	7	31.54
3	86-2-2	61	10	31.9	65	10	23.83	146	77-7-10	73	5	29.3	74	7	30.34
4	104-1-10	61	7	34.0	61	5	36.42	147	24-3-9	73	7	28.5	57	5	31.26
5	114-3-3	61	5	27.1	61	8	26.12	148	126-1-3	73	6	28.9	69	8	29.37
6	24-4-10	61	8	32.1	59	5	32.25	149	48-1-7	73	6	34.1	71	4	38.8
7	110-2-1	62	5	26.0	84	4	25.77	150	89-2-3A4	73	5	30.2	69	7	28.99
8	24-3-7	62	5	30.2	57	5	31.74	151	138-31	73	4	30.7	76	6	30.4
9	114-1-6	62	6	29.6	67	4	29.96	152	96-5	73	4	30.2	82	5	29.9
10	63-1-6	62	6	31.1	64	7	38.85	153	128-4-5	73	3	31.1	72	3	31.24
11	55-1-5	63	13	28.4	58	6	27.67	154	138-5-3	74	4	28.3	71	5	29
12	128-1-1	63	6	36.3	101	6	34.17	155	55-1-7	74	10	17.8	74	13	25.32
13	104-2-8	64	7	31.4	76	6	35.8	156	24-3-3	74	8	28.9	69	8	31.81
14	28-1-1	64	7	26.9	56	10	26.84	157	96-4	74	13	30.0	84	7	40.68
15	003-2-2	64	10	29.9	60	8	30.05	158	27-2	74	7	34.2	78	8	35.16
16	86-2-1	64	6	26.9	69	5	45.2	159	86-1-8A9	74	5	21.9	64	6	24.82
17	38-2-3	64	4	33.2	61	5	30.04	160	97-1-2A6	74	6	27.2	73	6	26.43
18	55-3-9	65	4	28.3	66	5	30.73	161	107-2	74	6	30.3	82	7	28.24
19	55-4-1	65	7	29.1	59	5	30.66	162	110-2-3	75	7	25.8	83	7	26.01
20	116-2-4	65	5	31.1	74	3	28.76	163	53-1-9	75	15	-	89	8	27.48
21	104-2-1	65	5	25.6	69	4	38.25	164	34-1-4	75	4	30.1	91	6	41.2
22	63-5-2	65	7	29.8	60	4	31.83	165	94-1-1	75	11	39.1	73	5	28.25
23	63-2	65	2	34.0	60	4	34.9	166	151-2-8	75	10	25.4	84	5	24.33
24	121-2-6	65	6	29.1	79	7	28.13	167	61-4-1	75	7	25.3	71	4	35.01
25	121-5-3	65	16	29.1	83	7	28.75	168	94-1-10	75	6	32.0	70	7	27.82
26	55-2-4	65	8	32.3	60	5	36.74	169	73-2	75	3	36.4	57	7	35.21
27	39-2-2	66	4	25.5	59	5	35.36	170	114-1-2A3	75	5	30.6	64	6	29.07

28	39-1-1A10	66	9	29.7	62	10	30.45	171	151-1-9	76	9	32.5	73	4	33.49
29	94-2-1	66	5	33.2	57	4	34.3	172	100-1-8	76	5	29.4	71	4	30.02
30	005-3-10	66	8	29.6	90	5	28.73	173	117-3-2	76	8	32.7	66	6	31.88
31	100-2-1	66	16	27.0	94	5	25.11	174	114-1-10	76	3	32.5	55	7	34.42
32	36-1-1A3	66	4	29.0	75	3	33.77	175	121-1-3	76	7	30.1	69	7	29.94
33	97-3-2A3	66	6	27.2	71	7	36.17	176	126-1-10	76	10	31.8	82	9	35.6
34	63-3-9	66	4	34.2	60	5	35.5	177	55-4-7	76	3	28.7	71	6	28.48
35	85-1-1	66	13	29.7	71	7	31.4	178	14-2-10	76	4	29.6	72	5	29.47
36	117-3-1	67	3	30.1	71	5	40.6	179	46-2-10	77	4	26.8	67	5	27.82
37	40-1-4	67	5	28.7	68	9	31.28	180	55-2-1	77	4	34.7	82	3	34.67
38	114-3-1	67	8	27.0	76	9	27.49	181	77-5-4	77	4	15.7	65	5	29.98
39	61-1-9	67	6	30.8	71	3	27.95	182	107-6	77	8	28.1	88	3	34.35
40	003-2-7	67	10	29.4	81	12	26.66	183	38-2-2	77	6	34.3	90	8	36
41	128-1-4	67	6	39.1	67	6	35.7	184	138-31-3	77	4	30.2	73	4	30.6
42	46-2-5	67	6	28.5	74	7	28.33	185	48-3-8	77	6	33.9	73	6	34.6
43	138-2-2	67	4	30.1	71	5	30.12	186	121-2-7	78	12	28.7	82	12	35.11
44	48-2-2A8	67	5	30.2	70	5	31.36	187	114-1-1A3	78	3	37.7	88	4	28.92
45	63-4-1	67	5	35.3	61	3	42.04	188	77-1-4A5	78	9	30.1	89	11	28.98
46	46-2-4	67	9	32.1	61	4	29.2	189	89-1-7	78	7	29.0	67	5	28.81
47	114-3-5	67	7	34.5	69	7	31.23	190	89-3-4	78	6	32.2	71	4	29.62
48	63-6-5	67	5	30.6	71	4	32.25	191	55-3-3	78	2	30.4	59	5	30.13
49	36-3-1	67	7	29.2	59	7	28.78	192	117-2-5	79	4	28.9	83	6	38.68
50	63-1-2	67	4	35.1	69	4	32.08	193	94-3-3	79	7	29.2	71	5	29.26
51	97-4-3A10	67	8	31.2	63	7	30.87	194	140-1-1	79	7	35.7	73	4	33.41
52	121-2-1	67	9	26.3	71	7	28.85	195	48-2-4A8	79	6	28.3	76	4	30.13
53	39-1-7	67	4	26.9	73	5	29.25	196	77-2-8	79	7	28.8	72	4	27.61
54	63-5-4	67	4	31.9	72	4	33.15	197	101-2	79	6	33.9	69	4	33.24
55	34-1-2	67	6	28.0	71	3	28.29	198	96-2-6	79	6	32.9	76	6	31.88
56	115-1-4	67	12	27.8	60	8	30.44	199	94-3-5	79	5	29.7	72	6	32.68
57	63-3-2	67	4	30.3	73	5	38.2	200	48-3-6	79	4	33.4	79	7	33.66
58	48-3-7	68	6	30.9	60	5	32.92	201	94-5-3	79	6	31.9	71	5	32.5
59	24-3-6	68	12	29.5	61	4	29.69	202	128-4-4	79	4	29.0	71	3	29.24

60	24-3-4	68	10	30.0	59	4	29.03	203	48-3-1	80	4	33.1	81	6	31.84
61	77-4-10	68	9	28.5	61	5	28.95	204	143-4-A4	80	7	27.4	76	5	28.72
62	14-4	68	8	31.3	71	6	34.35	205	117-1-2	80	5	25.8	68	4	27.63
63	14-2-7	68	13	25.8	83	15	28.24	206	151-2-3	80	7	31.2	84	4	31.6
64	104-1-8	68	6	38.4	71	4	36.68	207	104-3-5	80	25	31.7	73	4	28.97
65	114-1-8	68	7	36.1	74	4	31.85	208	96-2	80	4	29.6	80	8	31.84
66	140-1-4	68	21	28.4	64	8	27.67	209	83-1-7	81	10	27.2	70	5	25.58
67	61-1-10	69	7	39.5	69	4	31.4	210	50-2-4	81	7	32.5	75	6	30
68	77-4-1	69	4	28.5	63	9	29.02	211	89-1-1	81	14	26.6	79	5	26.63
69	20-1-6	69	10	31.2	71	5	29.96	212	003-2-6	81	10	28.0	85	10	27.46
70	77-1-1A5	69	19	28.9	70	6	26.96	213	63-1-10	81	5	38.3	87	3	33.09
71	128-1-5	69	10	32.0	60	6	33.92	214	128-2	81	8	30.3	71	6	31.7
72	89-1-10	69	7	28.7	63	6	28.27	215	104-4-6	81	6	38.7	74	5	30.28
73	61-1-4	69	5	31.2	73	3	29.64	216	143-4-4A4	81	7	26.2	85	5	26.93
74	117-3-3	70	6	35.7	74	7	30.7	217	138-1	81	12	32.0	74	7	33.92
75	126-1-8	70	8	32.4	67	4	35.8	218	143-2-1	81	5	30.9	75	11	31.18
76	100-2-2	70	5	34.3	62	4	38.83	219	151-1-8	81	3	35.4	70	5	35.23
77	104-1-3	70	11	30.0	69	4	30	220	151-2-10	81	7	35.0	86	7	32.1
78	86-1-6A9	70	9	28.0	73	7	28.16	221	94-3-9	82	6	28.3	67	5	33.41
79	001-10	70	9	28.7	96	6	27.4	222	83-1-8	82	4	24.7	65	8	28.21
80	77-3	70	7	35.2	72	7	34	223	005-7-1	82	11	27.0	85	13	26.91
81	140-1-6	70	5	29.2	63	4	29.76	224	38-2-4	82	8	26.7	91	6	23.72
82	97-4-1A10	70	5	30.6	71	6	30.51	225	35-1-1	82	6	34.0	75	6	38.2
83	63-6-4	70	8	30.7	74	5	27	226	128-4-2	82	24	30.1	73	12	28
84	77-1-2A5	70	11	27.2	60	5	29.03	227	46-3-3A3	83	8	30.2	73	3	91.78
85	46-2-2	70	7	29.9	66	6	28.5	228	20-1-5	83	5	24.4	82	7	24.51
86	121-1-7	70	6	32.5	60	5	30.6	229	116-2-2	83	8	28.2	90	9	27.54
87	126-1-1	70	7	28.2	77	7	31.65	230	35-1-2	83	6	35.5	85	5	31.54
88	63-3-1	70	5	36.4	61	6	33.67	231	117-2-6	83	11	29.5	76	9	37.88
89	55-1-10	70	7	34.0	61	5	36.6	232	117-1-3	83	5	31.1	88	4	30.39
90	115-1-5	70	5	28.7	78	8	25.76	233	77-7-7	83	9	30.1	76	6	26.31
91	77-5-9	70	5	30.1	69	5	31.14	234	126-1-6	83	4	31.6	84	6	31.18
92	128-4-6	70	5	26.1	61	3	35.68	235	63-6-3	84	9	26.4	90	8	31.2

93	43-1-2A3	70	5	29.3	78	8	27.74	236	116-1-9	84	10	33.1	62	5	33.82
94	151-4-1	70	15	30.4	78	6	28.84	237	83-2-1	84	5	36.7	74	5	26.33
95	77-1-3A5	70	12	29.5	86	12	27.65	238	151-3-3	84	5	31.5	87	7	30.57
96	94-4-3A7	70	5	30.8	79	7	23.36	239	83-1-10	84	8	24.6	83	5	25.3
97	27-1-8	71	8	31.7	71	7	32.14	240	53-1-1	84	10	24.8	96	6	22.98
98	77-2-4	71	4	36.7	72	4	36.36	241	34-5-2	84	4	40.3	79	5	44.91
99	89-2-1A4	71	8	27.4	67	6	28.65	242	116-1-3	84	3	31.3	71	3	30.5
100	77-6-2	71	11	26.0	80	19	28.12	243	61-3	84	4	26.4	62	3	31.94
101	128-1-6	71	7	26.9	60	6	38.6	244	48-1-6	84	4	30.7	73	4	50.63
102	48-1-5	71	5	31.8	71	5	32.25	245	50-1-1	84	10	22.9	102	4	28.74
103	61-1-1	71	5	24.5	73	4	23.98	246	**117-1-5**	84	2	26.2	82	4	28.99
104	128-4-9	71	3	33.2	59	5	32.28	247	27-5-9	85	7	34.9	81	6	33.33
105	87-4	71	5	33.8	60	4	34.6	248	35-1-10	85	6	36.8	89	2	45.26
106	117-2-4	71	5	31.0	73	6	33.07	249	48-1-8	85	4	34.1	61	9	39.99
107	77-5-3	71	12	30.5	66	5	31.42	250	50-2-3	85	8	32.6	75	6	36.8
108	0003-1-02	71	4	29.0	66	6	37.58	251	61-1-5	85	7	31.0	64	6	31.91
109	77-7-5	71	5	28.9	59	6	30.33	252	77-2-2	85	6	29.3	71	4	28.3
110	46-2-1	71	6	31.2	66	5	32.6	253	151-1-10	85	13	22.1	88	6	22.71
111	114-3-4	71	7	29.0	81	9	28.86	254	151-4-4	85	6	34.0	63	5	32.36
112	003-2-8	71	5	32.2	74	5	31.06	255	96-2-1	85	4	32.3	92	5	32.01
113	27-1-4	71	5	34.5	71	7	32.67	256	143-2-8	86	9	36.7	74	8	34.87
114	77-7-6	71	9	28.3	71	6	28.72	257	03-1-001	86	8	27.5	80	8	25.58
115	128-4-7	71	5	34.2	70	4	30.32	258	116-4-2A6	86	6	30.5	90	3	26.95
116	35-1-7	72	7	37.3	75	4	42.41	259	138-2-4	86	17	31.2	83	4	29.64
117	24-1	72	6	28.2	71	5	32.64	260	46-4-1A4	86	4	30.7	87	5	28.75
118	48-2-3A8	72	7	31.4	70	4	27.66	261	117-4-2A5	87	16	35.1	72	7	35.8
119	104-4-7	72	8	29.6	72	5	29.84	262	151-3-2	87	13	32.5	72	8	44.8
120	104-3-6	72	10	28.5	69	8	29.96	263	34-1-1	87	6	28.7	88	5	28.5
121	27-5-5	72	4	30.5	73	6	34.8	264	116-4-5A6	87	6	30.8	75	6	29.72
122	117-4-3A5	72	8	30.2	71	6	33.53	265	151-1-6	87	24	25.8	73	6	19.92
123	94-2-3	72	-	21.3	89	10	21.35	266	97-1-1A6	87	14	25.7	71	10	24.52

124	27-1-6	72	6	38.3	68	7	29.1	267	24-4-9	87	7	32.5	89	6	31.88
125	94-1-5	72	5	32.5	67	4	33.8	268	53-1-5	87	7	34.1	96	4	30.47
126	27-5-4	72	5	29.3	73	6	31.6	269	46-3-3	87	3	31.1	85	3	31.05
127	55-1-9	72	5	34.0	76	5	33.8	270	39-2-1	87	6	27.6	88	5	24.51
128	126-1-7	72	5	27.0	67	5	28.2	271	110-1-5	88	27	26.4	96	10	25.12
129	94-4-2A7	72	19	28.5	76	13	44.3	272	151-2-5	88	4	34.6	107	6	31.5
130	89-3-2	72	6	27.5	70	6	30.1	273	55-3-8	88	5	33.6	100	4	36.91
131	97-2-6	72	24	31.6	57	10	29.06	274	151-3-6	88	6	30.2	90	5	31
132	97-2-5	72	5	23.9	71	7	33.55	275	143-2-10	88	3	38.3	90	5	97.32
133	128-4-8	72	3	34.6	61	5	35.57	276	151-3-8	89	6	29.0	80	10	28.43
134	63-3-10	72	4	30.9	58	5	29.89	277	117-4-1A5	90	15	25.8	100	13	22.91
135	24-4-4	72	6	34.8	83	6	27.16	278	138-5-5	91	4	34.1	0	5	24.56
136	48-3-3	72	5	30.7	81	4	31.05	279	151-1-2	91	4	31.4	101	6	29.67
137	48-2-5A8	73	6	30.5	62	6	32.5	280	143-2-9	94	13	34.7	81	6	33.51
138	39-1-6	73	11	28.7	66	4	25.54	281	138-31-2	97	3	30.5	87	4	25.94
139	121-2-4	73	7	34.5	72	6	32.26	282	151-2-2	97	13	36.1	100	6	28.5
140	94-2-4	73	7	34.3	63	4	33.62	283	89-1-8	100	3	28.4	71	8	29.66
141	143-2-7	73	7	32.5	67	6	29.1	284	138-5-4	102	8	29.9	93	5	28.85
142	94-5-6	73	8	31.5	74	5	31.02	285	143-2-4	110	11	34.9	86	12	35
143	34-1-3	73	14	26.9	80	10	28.47								

Three traits were included, heading date (Hd), tiller number at maturity (T) and 1000-grain weight (TGW), provided by Africa Rice Center (AfricaRice Research Station in Bouake (Cote d'Ivoire); http://www.africarice.org).

Table S10. Individuals of low- and high-value of Hd, T and TGW traits used in NGS-based BSA.

No	Name	Hd F9	Hd F10	Hd Mean	No	Name	Mean T F9	Mean T F10	Till Mean	No	Name	TGW F9	TGW F10	TGW Mean
		15 BILs lowest in heading date					15 BILs lowest in tiller					15 BILs lowest in TGW		
1	24-3-7	62	57	60	1	117-1-5	2	4	3	1	94-2-3	21.3	21.4	21.3
2	97-2-4	60	60	60	2	63-2	2	4	3	2	55-1-7	17.8	25.3	21.6
3	24-4-10	61	59	60	3	116-1-3	3	3	3	3	151-1-10	22.1	22.7	22.4
4	28-1-1	64	56	60	4	128-4-5	3	3	3	4	151-1-6	25.8	19.9	22.8
5	55-1-5	63	58	61	5	114-1-1A3	3	4	3	5	86-1-8A9	21.9	24.8	23.4
6	104-1-10	61	61	61	6	36-1-1A3	4	3	3	6	53-1-1	24.8	23	23.9
7	114-3-3	61	61	61	7	46-3-3	3	3	3	7	61-1-1	24.5	24	24.3
8	94-2-1	66	57	62	8	138-31-2	3	4	4	8	117-4-1A5	25.8	22.9	24.3
9	003-2-2	64	60	62	9	55-3-3	2	5	4	9	20-1-5	24.4	24.5	24.5
10	55-4-1	65	59	62	10	63-5-4	4	4	4	10	151-2-8	25.4	24.3	24.9
11	38-2-3	64	61	63	11	35-1-10	6	2	4	11	83-1-10	24.6	25.3	25
12	63-5-2	65	60	63	12	63-1-2	4	4	4	12	97-1-1A6	25.7	24.5	25
13	63-2	65	60	63	13	48-1-6	4	4	4	13	38-2-4	26.7	23.7	25.2
14	55-2-4	65	60	63	14	128-4-8	3	5	4	14	110-2-1	26	25.8	26
15	39-2-2	66	59	63	15	138-31-3	4	4	4	15	110-2-3	25.8	26	26
		15 BILs highest in heading date					15 BILs highest in tiller					15 BILs highest in TGW		
1	116-4-2A6	86	90	88	1	003-2-7	10	12	11	1	117-4-2A5	35.1	35.8	35.5
2	24-4-9	87	89	88	2	143-2-4	11	11.5	12	2	73-2	36.4	35.21	35.8
3	96-2-1	85	92	89	3	55-1-7	10	13.2	12	3	143-2-8	36.7	34.87	35.8
4	151-3-6	88	90	89	4	34-1-3	14	9.5	12	4	35-1-1	34	38.2	36.1
5	143-2-10	88	90	89	5	005-7-1	11	12.9	12	5	48-1-7	34.1	38.8	36.5
6	53-1-1	84	96	90	6	77-1-3A5	12	11.9	12	6	77-2-4	36.7	36.36	36.5
7	53-1-5	87	96	92	7	97-1-1A6	14	10.4	12	7	100-2-2	34.3	38.83	36.5
8	110-1-5	88	96	92	8	121-2-7	12	12	12	8	48-1-8	34.1	39.99	37
9	138-31-2	97	87	92	9	117-4-1A5	15	12.6	14	9	128-1-4	39.1	35.7	37.4
10	55-3-8	88	100	94	10	14-2-7	13	15	14	10	104-1-8	38.4	36.68	37.5
11	117-4-1A5	90	100	95	11	77-6-2	11	19.3	15	11	151-3-2	32.5	44.8	38.6
12	151-1-2	91	101	96	12	94-4-2A7	19	12.9	16	12	63-4-1	35.3	42.04	38.7
13	151-2-5	88	107	98	13	97-2-6	24	9.8	17	13	35-1-7	37.3	42.41	40
14	138-5-4	102	93	98	14	128-4-2	24	11.8	18	14	35-1-10	36.8	45.26	41
15	151-2-2	97	100	99	15	110-1-5	27	9.6	18	15	34-5-2	40.3	44.91	42.6

Hd: Heading date, **T:** tiller number at maturity, **TGW:** 1000-grain weight, **F9&10:** Generation 9&10, **BILs:** Backcross Inbred Lines. **2:** Illustration of A) Association analysis, and B) Investigation of candidate genes.
